# RNA-Seq-based transcriptome analysis of methicillin-resistant *Staphylococcus aureus* growth inhibition by propionate

**DOI:** 10.3389/fmicb.2022.1063650

**Published:** 2022-12-22

**Authors:** Jintaek Im, Dongwook Lee, Ok-Jin Park, Sathishkumar Natarajan, Junhyung Park, Cheol-Heui Yun, Seung Hyun Han

**Affiliations:** ^1^Department of Oral Microbiology and Immunology, and Dental Research Institute, School of Dentistry, Seoul National University, Seoul, South Korea; ^2^3BIGS Co., Ltd., Hwaseong, South Korea; ^3^Department of Agricultural Biotechnology, Research Institute of Agriculture and Life Sciences, Seoul National University, Seoul, South Korea; ^4^Institutes of Green Bio Science and Technology, Seoul National University, Pyeongchang, South Korea

**Keywords:** short-chain fatty acids, propionate, bacteriostatic, RNA-Seq, metabolic pathway

## Abstract

*Staphylococcus aureus* is a pathogen that causes a variety of infectious diseases such as pneumonia, endocarditis, and septic shock. Methicillin-resistant *S. aureus* (MRSA) evades virtually all available treatments, creating the need for an alternative control strategy. Although we previously demonstrated the inhibitory effect of sodium propionate (NaP) on MRSA, the regulatory mechanism of this effect remains unclear. In this study, we investigated the regulatory mechanism responsible for the inhibitory effect of NaP on MRSA using RNA-Seq analysis. Total RNAs were isolated from non-treated and 50 mM NaP-treated S. aureus USA300 for 3 h and transcriptional profiling was conducted by RNA-Seq analysis. A total of 171 differentially expressed genes (DEGs) with log_2_ fold change ≥2 and *p* < 0.05 was identified in the NaP treatment group compared with the control group. Among the 171 genes, 131 were up-regulated and 40 were down-regulated. Upon gene ontology (GO) annotation analysis, total 26 specific GO terms in “Biological process,” “Molecular function,” and “Cellular component” were identified in MRSA treated with NaP for 3 h. “Purine metabolism”; “riboflavin metabolism”; and “glycine, serine, and threonine metabolism” were identified as major altered metabolic pathways among the eight significantly enriched KEGG pathways in MRSA treated with NaP. Furthermore, the MRSA strains deficient in *purF*, *ilvA*, *ribE*, or *ribA*, which were the up-regulated DEGs in the metabolic pathways, were more susceptible to NaP than wild-type MRSA. Collectively, these results demonstrate that NaP attenuates MRSA growth by altering its metabolic pathways, suggesting that NaP can be used as a potential bacteriostatic agent for prevention of MRSA infection.

## Introduction

*Staphylococcus aureus* is one of the most frequently isolated human Gram-positive pathogens that causes a variety of human diseases such as pneumonia, gastroenteritis, endocarditis, and septic shock (Lowy, 1998; Alberti et al., 2002). Since *S. aureus* can readily develop or obtain resistance to antibiotics, emergence of antibiotic-resistant *S. aureus* is a growing problem in human healthcare as treatment failures are associated with not only risk of the life but also enormous medical cost (Graves et al., 2010; Vestergaard et al., 2019). Methicillin-resistant *S. aureus* (MRSA) is the most prevalent such pathogen, with persistently high morbidity and mortality (Turner et al., 2019). In the United States, total of 119,247 cases of *S. aureus* infections, including MRSA, with 19,832 deaths had been reported in 2017 (Kourtis et al., 2019). Community-acquired MRSA (CA-MRSA) has rapidly increased the global incidence of *S. aureus* infection and *S. aureus* USA300 is known as one of the most predominant CA-MRSA strain worldwide (Planet, 2017). Furthermore, *S. aureus* USA300 is considered as an epidemic strain leading to severe infections and outbreaks due to its relatively higher virulence and invasive capacity compared with other CA-MRSA strains (Selan et al., 2018). Vancomycin and daptomycin are used as first-line treatment options for MRSA infection (Liu et al., 2011), but these antibiotic treatments have limitations including low tissue absorption, slow bactericidal efficacy, and antibiotic resistance (Kullar et al., 2013; Moise et al., 2013; Ortwine and Bhavan, 2018). Moreover, since an *S. aureus* vaccine is not currently available for preventing infection (Clegg et al., 2021), a novel strategy to effectively control MRSA infection, especially USA300 strain, is urgently needed.

Short-chain fatty acids (SCFAs) are small organic monocarboxylic acids that are mainly produced by commensal bacteria in the colon through fermentation of dietary fibers and carbohydrates (Silva et al., 2020). Although roughly 500–600 mM of SCFAs are produced in the human gut per day, their amount and distribution can change depending on the fiber content in the diet and microbiota composition (Macfarlane and Macfarlane, 2003). Acetate (C2), propionate (C3), and butyrate (C4) are representative SCFAs accounting for more than 95% of all SCFAs; and their molar ratio in the human colon is about 60:20:20 (Cummings et al., 1987; Fernandes et al., 2014; Luu et al., 2019). SCFAs are utilized as energy sources by certain bacteria. For instance, *Treponema* species in dental plaque utilize butyrate produced by *Prevotella* and *Porphyromonas* species as their energy source (Hojo et al., 2009). Furthermore, SCFAs can regulate host immune responses in various ways. For example, butyrate suppresses *S. aureus* lipoprotein-induced nitric oxide production in macrophages (Park et al., 2019). In addition, butyrate and propionate inhibit the activation of antigen-specific CD8^+^ T lymphocytes by suppressing IL-12 production in dendritic cells (Nastasi et al., 2017).

Previous studies have reported antimicrobial effects of SCFAs on various pathogenic bacteria. For instance, butyrate inhibited the growth of *Helicobacter pylori* by impairing integrity of the cell envelope (Yonezawa et al., 2012) while acetate suppressed the growth of *Escherichia coli* by interfering with its methionine biosynthesis (Roe et al., 2002) or perturbing acetyl phosphate concentration needed for sugar uptake (Pinhal et al., 2019). In addition, propionate decreased the growth of clinically isolated *Enterococcus faecalis* (Jeong et al., 2019b). Consistent with these observations, our previous study demonstrated a bacteriostatic effect of propionate on MRSA (Jeong et al., 2019a). In that study, propionate was more effective at attenuating bacterial growth than acetate and butyrate. Moreover, propionate effectively alleviated MRSA infection by inhibiting bacterial growth in a murine skin infection model. Although these findings suggest propionate as an alternative strategy to treat MRSA infection, underlying mechanisms have not been fully characterized.

Recently, development of transcriptomics based on next-generation sequencing allows quantitative analysis of the expression of numerous genes simultaneously and consequential comparative analysis (Westermann and Vogel, 2021). Among transcriptomics methods, RNA sequencing (RNA-Seq) permits meticulous evaluation of transcription levels compared with other methods (Westermann and Vogel, 2021). Thus, we conducted transcriptomic analysis to characterize gene alterations to identify the underlying mechanisms of the bacteriostatic effect of propionate on MRSA.

## Materials and methods

### Reagent and chemicals

Sodium propionate (NaP) was purchased from Sigma-Aldrich Inc. (St. Louis, MO, United States). NaP was dissolved in endotoxin-free distilled water and filtered using a syringe filter (0.2 μm pore size; Corning, Corning, NY, United States). Trypticase soy broth (TSB) and Bacto agar were purchased from BD Biosciences (Franklin Lakes, NJ, United States).

### Bacterial growth

*Staphylococcus aureus* USA300 was used as a representative MRSA strain, and its wild-type and mutant strains deficient for the *purF* (*∆purF*), *ilvA* (*∆ilvA*), *ribE* (*∆ribE*), or *ribA* (*∆ribA*) gene were obtained from the Nebraska Transposon Mutant Library (Omaha, NE, United States). A single colony of each strain of *S. aureus* USA300 grown on a TSB agar plate (TSB broth containing 1.5% Bacto agar) was cultured in TSB broth at 37°C overnight, and 1% of the culture was inoculated into fresh TSB broth in the presence or absence of 50 mM NaP on a flat-bottom 96-well plate (Thermo Scientific, Waltham, MA, United States). The bacteria were cultured at 37°C under aerobic shaking (180 rpm/min) culture conditions, and the bacterial growth was estimated by measuring optical density at 600 nm every 1 or 2 h up to 12 h using a microplate reader (SPARK 10 M, Tecan, Zurich, Swiss).

### Minimum inhibitory concentration and minimum bactericidal concentration test

The minimum inhibitory concentration (MIC) test was performed as previously described (Jeong et al., 2019a). Briefly, 1% of the overnight cultured *S. aureus* USA300 was inoculated into fresh TSB broth containing serially diluted (0, 3.9, 7.8, 15.6, 31.3, 62.5, 125, 250, or 500 mM) NaP on a flat-bottom 96-well plate. The bacteria were cultured at 37°C for 24 h under aerobic shaking culture conditions, and optical density at 600 nm was measured using a microplate reader (SPARK, Tecan). MIC was defined as the minimum concentration of NaP needed for non-visible growth of *S. aureus* USA300 at 24 h. To determine minimum bactericidal concentration (MBC), the bacterial cultures showing low or no bacterial growth on the MIC test were inoculated into fresh TSB broth and incubated at 37°C for 24 h under aerobic shaking culture conditions. Bacterial growth was examined by measuring optical density at 600 nm (SPARK).

### Extraction and purification of RNA

One percent of the overnight cultured *S. aureus* USA300 was inoculated into fresh TSB broth containing 50 mM NaP in 50 ml conical tubes (SPL Life Science, Gyeonggi-Do, Republic of Korea) and cultured at 37°C for 3 h under aerobic shaking culture conditions. The bacteria were harvested by centrifugation at 8,000 *× g* for 10 min, and bacterial pellets were suspended in phosphate-buffered saline (PBS) containing 100 μg/ml of lysostaphin (Sigma-Aldrich Inc.). After incubation at 37°C for 15 min, the pellets were collected by centrifugation at 8,000 *× g* for 10 min; and total RNA was isolated and purified using a RNeasy^®^ Mini kit (Qiagen, Gaithersburg, MD, United States) according to the manufacturer’s instructions. The purified total RNA was used to construct complementary DNA (cDNA) libraries for RNA-Seq.

### Construction of a cDNA library and RNA-Seq

The construction of cDNA library and RNA-Seq data were performed by Macrogen (Seoul, Republic of Korea). The mRNA in the prepared total RNA was initially converted into a cDNA library using the Illumina^®^ TruSeq^™^ stranded mRNA sample prep kit (Illumina Inc., San Diego, CA, United States) according to the manufacturer’s guidance. In brief, bacterial rRNA was removed using the NEBNext^®^ rRNA depletion kit (NEB, Ipswich, MA, United States). The purified mRNA was fragmented into small pieces using divalent cations under elevated temperature. The cleaved RNA fragments were transcribed into first-strand cDNA using SuperScript II reverse transcriptase (Invitrogen, Waltham, MA, United States) and random primers. Then, the cDNA was synthesized to second-strand cDNA using DNA polymerase I, RNase H, and dUTP. Finally, single adenine bases were added to the cDNA fragments using a 3′ to 5′ exonuclease, and these adenylated cDNA fragments were ligated with adapters. The products were purified and enriched by PCR to create the cDNA library. The cDNA libraries were quantified using a KAPA library quantification kit (KAPA Biosystems, Wilmington, MA, United States) according to the qPCR quantification protocol guide (KAPA Biosystems) and quantified using the Agilent D1000 ScreenTape System (Agilent Technologies, Santa Clara, CA, United States). The resulting cDNA libraries (three libraries per treatment group) were sequenced on the NovaSeq 6,000 platform (Illumina Inc.).

### Identification and annotation of differentially expressed genes

The quality of raw data was checked by the FastQC.^1^ Trimmomatic (v.0.38) was then applied to examine adaptor contamination and to remove low-quality reads (Bolger et al., 2014). The clean reads were aligned onto the reference genome of *S. aureus* subsp. aureus USA300_FPR3757 (NCBI Reference Sequence: NC_007793.1) using HISAT2 (2.1.0) aligner (Kim et al., 2015). In addition, the abundance of mapped reads was counted and measured by fragments per kilobase of exon per million fragments mapped (FPKM; Anders et al., 2015). For analysis of differentially expressed genes (DEGs), the log2 values (FPKM) were initially calculated and normalized by quantile normalization to reduce systematic bias. Statistical analysis between treatment groups was conducted by independent t-test with the mean FPKM value for each gene, and genes with a fold-change ≥2 or ≤−2 with *p* < 0.05 were assigned as DEGs. All statistical analysis for DEGs was performed using the DESeq2 R package (v.1.26.0; Anders and Huber, 2010). For functional analysis of DEGs, Kyoto encyclopedia of genes and genomes (KEGG) pathway enrichment analysis was conducted using a KEGG orthology-based annotation system (KOBAS; Bu et al., 2021), and gene ontology (GO) annotation analysis was performed using BLAST search against UniProt database (Ashburner et al., 2000). A *p*-value of <0.05 calculated by Fisher’s exact test was set as the cutoff criteria for the KEGG pathway enrichment analysis.

### Real-time PCR validation

To validate the RNA-Seq data, real-time PCR analysis was conducted to quantify the mRNA transcripts of eight randomly selected DEGs as previously described (Song et al., 2021) with minor modifications. Briefly, one microgram of total RNA isolated from *S. aureus* USA300 under identical processing conditions with those of the RNA-Seq samples was subjected to cDNA synthesis using random hexamers and reverse transcriptase (Promega, Madison, WI, United States). The mRNA expression was determined using the StepOnePlus real-time system (Applied Biosystems, Waltham, MA, United States) under these reaction conditions: initial denaturation at 95°C for 10 min, followed by amplification by 40 cycles of 95°C for 15 s, and 60°C for 1 min. In addition, 16S rRNA was used as an internal control to normalize the target gene mRNA expression. The information from 10 randomly selected DEGs and details of primers used in the current study are listed in Supplementary Table S1.

## Results

### Bacteriostatic effect of NaP on methicillin-resistant *Staphylococcus aureus*

**Figure 1:**
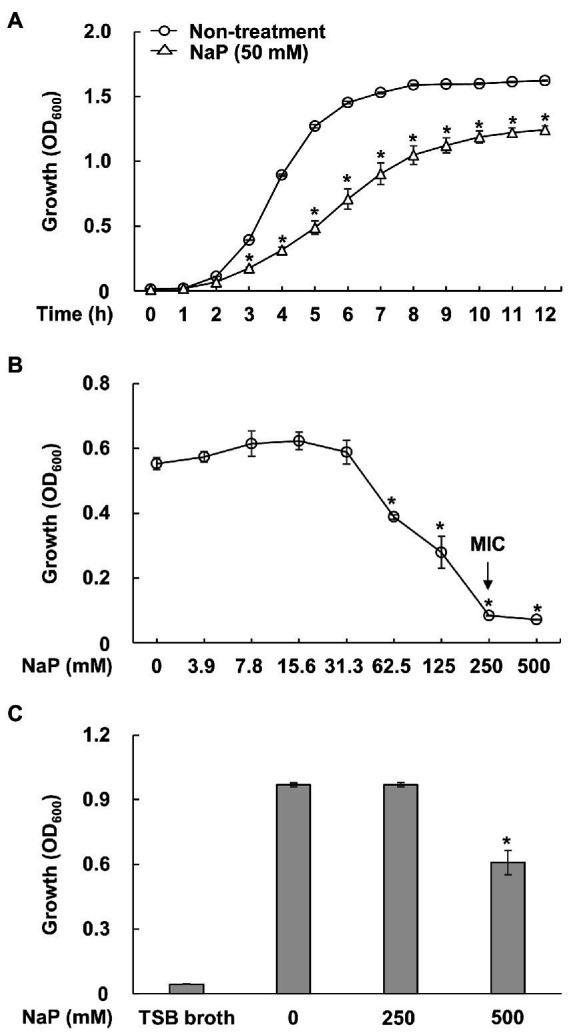
Bacteriostatic effect of NaP on MRSA. **(A)** *S. aureus* USA300 was cultured in the presence or absence of 50 mM NaP at 37°C for 12 h under aerobic shaking culture conditions. Bacterial growth was examined by measuring optical density at 600 nm hourly up to 12 h. Data shown are the mean value ± SEM of triplicate samples. ^*^p < 0.05 compared with the non-treatment group at each time point. **(B)** *S. aureus* USA300 was incubated in the presence of various concentrations of NaP at 37°C for 24 h under aerobic shaking culture conditions. After incubation, optical density at 600 nm was measured; the black arrows in the figure indicate the MIC of NaP. Data shown are the mean ± SEM of triplicate samples. ^*^p < 0.05 compared with the control group. **(C)** The bacteria cultures showing low or no bacterial growth on the MIC test were inoculated in fresh TSB broth and incubated at 37°C for 24 h under aerobic shaking culture conditions. The bacterial growth was then examined by measuring optical density at 600 nm. Data shown are the mean ± SEM of triplicate samples. ^*^p < 0.05 compared with the non-treatment group.

To confirm the growth inhibitory effect of NaP on MRSA as previously reported (Jeong et al., 2019a), growth of *S. aureus* USA300 in the presence or absence of 50 mM NaP was initially examined at the indicated time points under aerobic shaking culture conditions. As shown in Figure 1A, the growth inhibitory property of NaP was potent up to 12 h. Since the growth inhibition peaked at 6 h, RNA for RNA-Seq analysis was extracted 3 h post-treatment when initial growth inhibition by NaP was observed. To confirm that the inhibitory property of NaP on MRSA growth is bacteriostatic and not bactericidal, MIC and MBC tests were conducted. Since bacterial growth of *S. aureus* USA300 was not observed under 250 mM NaP treatment, this concentration was determined as the MIC of NaP (Figure 1B). Furthermore, an MBC test was conducted by examining the growth of bacterial cultures having low or no growth in the MIC test. Since bacterial growth was observed for all tested bacterial cultures even at 500 mM NaP, there was no MBC for NaP (Figure 1C). These results suggest that the inhibitory effect of NaP on MRSA growth is mediated by its bacteriostatic effect.

### Differentially expressed genes and their validation

**Figure 2:**
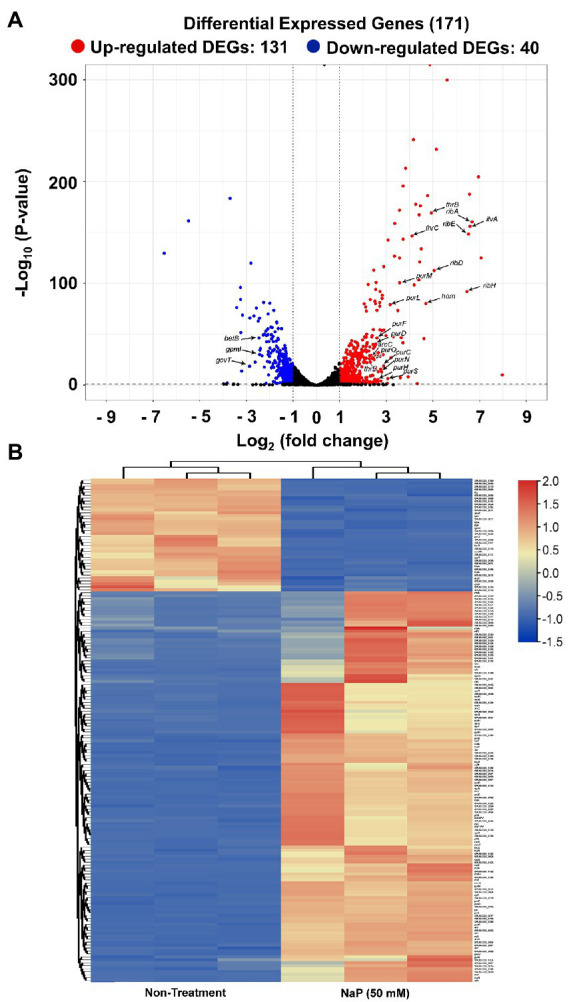
Differentially expressed genes in MRSA treated with NaP. The cDNA libraries were constructed from *S. aureus* USA300 incubated with or without 50 mM NaP for 3 h under aerobic shaking culture conditions. The libraries were subjected to RNA-Seq as described in the Materials and methods section. **(A)** Volcano plot of DEGs in the NaP-treated MRSA. Among the genes with log_2_ fold change ≥2 or ≤−2, up- and down-regulated genes at *p* < 0.05 in the NaP treatment group compared to non-treatment group were classified into DEGs and are represented by red and blue dots, respectively. **(B)** Heat map of the hierarchical clustering based on 171 DEGs (log_2_ fold change ≥2 or ≤−2, *p* < 0.05). Red and blue indicate up- and down-regulated DEGs in the NaP treatment group compared to the non-treatment group, respectively.

**Figure 3:**
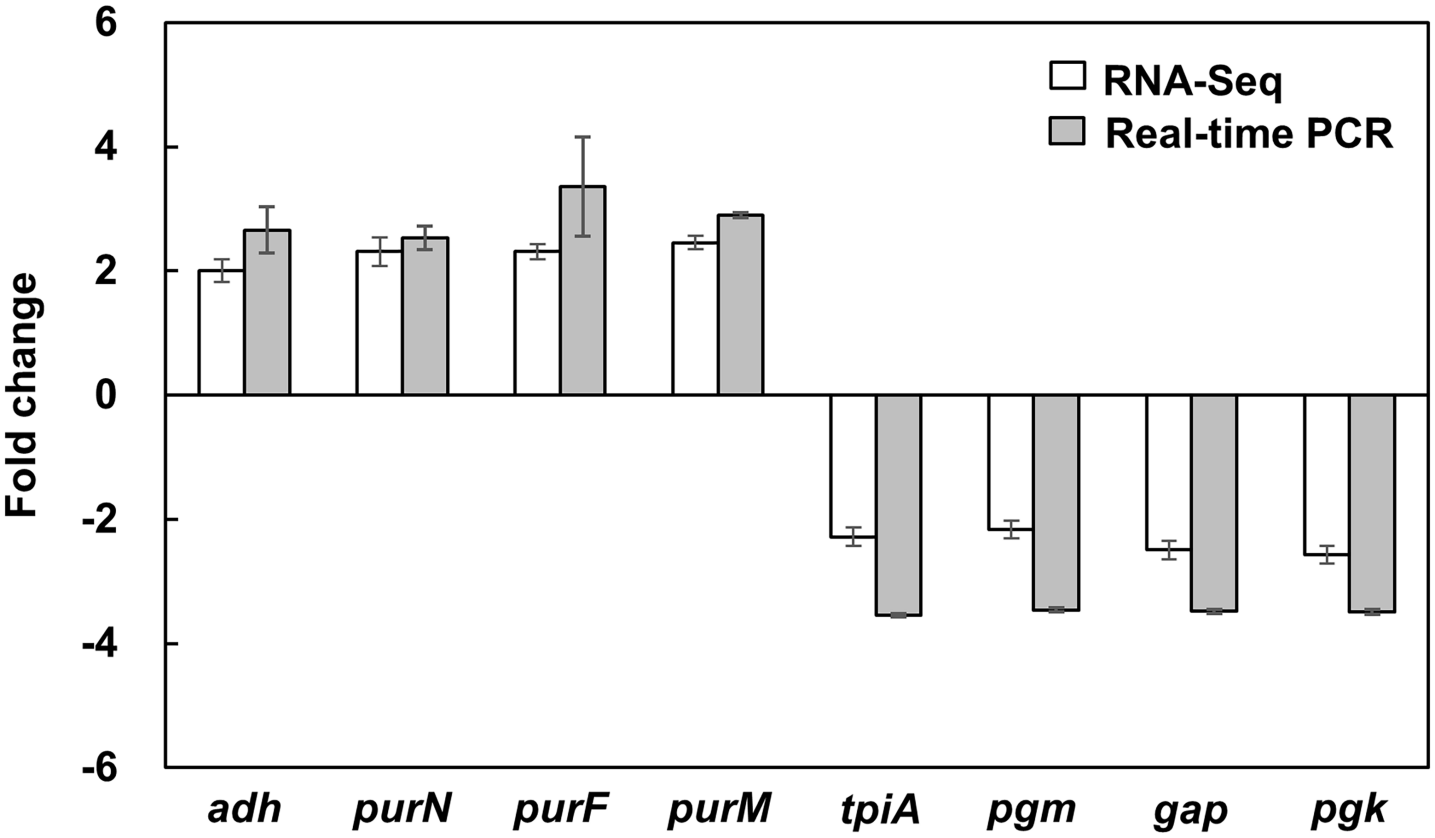
Validation of DEGs in MRSA treated with NaP using real-time PCR. *S. aureus* USA300 was cultured in the presence or absence of 50 mM NaP at 37°C for 3 h under aerobic shaking culture conditions. After incubation, total RNA was isolated and subjected to real-time PCR analysis for the eight randomly selected DEGs shown in Supplementary Table S1 as described in the Materials and methods. Data shown are the mean ± SEM of triplicate samples after normalization with 16S rRNA for each target gene.

To obtain a comprehensive analysis of the bacteriostatic effect of NaP on MRSA, six cDNA libraries were constructed from the NaP-treated and control *S. aureus* USA300, and each library was sequenced with Illumina NovaSeq 6,000 platform. RNA-Seq generated 31,085,806-31,782,620 raw reads per each cDNA library (a total of 188,252,040 from six libraries). For quality control, raw reads were confirmed using FastQC software (data not shown). Among them, 30,710,898-31,060,466 clean reads (99.17–99.29%) were generated from each sample after removing contaminated adaptor and low-quality sequences using Trimmomatic software (Table 1). Furthermore, we found that 99.48–99.64% of the clean reads were successfully aligned to the reference genome of *S. aureus* subsp. aureus USA300_FPR3757 (NCBI Reference Sequence: NC_007793.1). The NaP treatment group was compared with the control group (NT) to estimate the transcription profile of MRSA under NaP treatment. The selection criteria for DEGs were log_2_ fold change ≥2 or ≤−2 and *p* < 0.05. The expression of 171 genes was significantly different in the NaP treatment group compared with its control group. Among these, 131 genes were up-regulated, and 40 genes were down-regulated (Figure 2A). To examine a DEG profile under NaP treatment conditions, DEGs with similar metabolic functions were clustered by hierarchical clustering analysis using FPKM values. The clustering analysis showed that DEG expression patterns of MRSA were distinctly altered by NaP treatment (Figure 2B). The up- and down-regulated genes from the NaP treatment are listed in Supplementary Tables S2, S3, respectively. Furthermore, real-time PCR was conducted to validate the transcriptional profiles of RNA-Seq for the eight randomly selected DEGs *adh*, *purN*, *purF*, *purM*, *tpiA*, *pgm*, *gap*, and *pgk*. As shown in Figure 3, the DEG validation results were similar to those obtained from RNA-Seq analysis, suggesting that the DEGs were successfully identified by RNA-Seq.

**Table 1:** Summary of RNA-Seq alignment.

Features	NT1	NT2	NT3	NaP1	NaP2	NaP3
Raw Reads Number	31,283,942	31,085,806	31,509,084	30,967,610	31,622,978	31,782,620
Clean reads Number (%)	31,060,466 (99.29)	30,842,770 (99.22)	31,265,426 (99.23)	30,710,898 (99.17)	31,368,392 (99.19)	31,531,490 (99.21)
Total bases	3,159,678,142	3,139,666,406	3,182,417,484	3,127,728,610	3,193,920,778	3,210,044,620
GC%	34	34	34	34	34	34
Overall alignment with reference genome (%)	99.64	99.61	99.57	99.48	99.55	99.57

### Gene ontology annotation analysis

**Figure 4:**
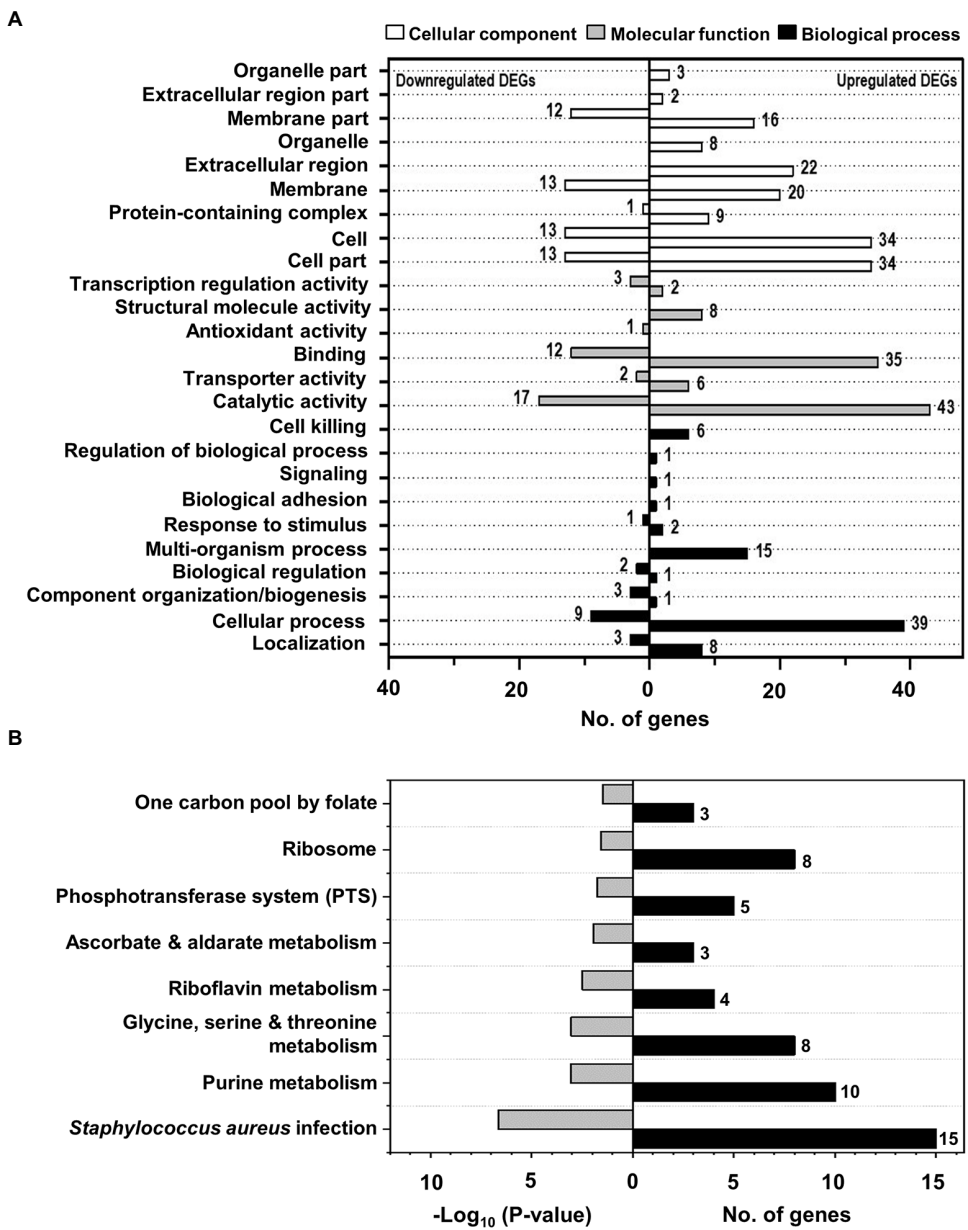
The identified GO terms and significantly enriched KEGG pathways of DEGs in MRSA treated with NaP. **(A)** GO annotation analysis for 171 DEGs was conducted using the UniProt GO database. The figure shows the identified 26 GO terms at subcategory levels of the three categories, including “cellular components,” “molecular function,” and “biological process,” with the numbers of up- and down-regulated DEGs belonging to each identified GO term. **(B)** KEGG pathway enrichment for 171 DEGs was performed using KOBAS, and significantly enriched pathways were identified by hypergeometric distribution at *p* < 0.01. The figure shows the eight most significantly enriched KEGG pathways with their *p*-values and the number of DEGs belonging to each enriched pathway.

To understand the function of the identified DEGs in the bacteriostatic effect of NaP on MRSA, GO annotation analysis was conducted for the 171 DEGs. For this, the DEGs were assigned to one or more GO terms and mainly categorized into three categories, including “cellular components,” “molecular function,” and “biological process” comprising 200, 129, and 93 subcategories, respectively. The total 26 GO terms at subcategory levels belonging to the three categories were identified and illustrated in Figure 4A. In the cellular components’ category, nine specific GO terms were identified in MRSA treated with NaP, including “organelle part,” “extracellular region part,” “membrane part,” “organelle,” “extracellular region,” “membrane,” “protein-containing complex,” “cell,” and “cell part.” In addition, six specific GO terms belonging to category of molecular function were annotated, such as “transcription regulation activity,” “structural molecule activity,” “antioxidant activity,” “binding,” “transporter activity,” and “catalytic activity.” On the other hand, total 10 specific GO terms composed of biological process were annotated, including “cell killing,” “regulation of biological process,” “signaling,” “biological adhesion,” “response to stimulus,” “multi-organism process,” “biological regulation,” “component organization and biogenesis,” “cellular process,” and “localization.” Details of the identified GO terms at subcategory levels together with a list of DEGs belonging to each GO term are presented in Supplementary Table S4.

### Kyoto encyclopedia of genes and genomes pathway enrichment

To further identify the involved pathways in the inhibition of MRSA growth by NaP treatment, DEGs were mapped to the KEGG database, and KEGG pathway enrichment analysis was performed. A total of eight pathways was significantly enriched: “one carbon pool by folate”; “ribosome”; “phosphotransferase system (PTS)”; “ascorbate and aldarate metabolism”; “riboflavin metabolism”; “glycine, serine, and threonine metabolism”; “purine metabolism”; and “*Staphylococcus aureus* infection” (Figure 4B; Supplementary Table S5; Supplementary Figure S1–S4). Since bacteriostatic agents mainly affect the metabolic state of the bacterium (Lobritz et al., 2015), we selected “purine metabolism”; “riboflavin metabolism”; and “glycine, serine, and threonine metabolism” as potent metabolic pathways responsible for the bacteriostatic effect of NaP on MRSA. Since it has been reported that purine metabolism plays a key role in cell wall synthesis which is closely related to changes in bacterial morphology (Sobral et al., 2007), we previously examined the effects of NaP on morphology of *S. aureus* USA300 by using scanning electron microscopy. The morphological changes, including bacterial size and membrane integrity, were not observed by NaP treatment when compared to those of non-treatment group (Jeong et al., 2019a). Furthermore, we examined the expression of genes in the selected pathways (Supplementary Figure S1). In the purine metabolic pathway, 9 of 10 genes encoding enzymes involved in purine biosynthesis were up-regulated by NaP treatment, including phosphoribosylamino imidazole-succinocarboxamide synthase (*purC*; EC: 6.3.2.6), phosphoribosylformyl glycinamidine synthase (*purS*; EC: 6.3.5.3), IMP cyclohydrolase (*purH*; EC: 2.1.2.3; 3.5.4.10), phosphoribosylglycinamide formyltransferase (*purN*; EC: 2.1.2.2), phosphoribosylamine-glycine ligase (*purD*; EC: 6.3.4.13), phosphoribosylformyl glycinamidine synthase II (*purL*; EC: 6.3.5.3), phosphoribosylformylglycinamidine synthase I (*purQ*; EC: 6.3.5.3), phosphoribosylaminoimidazole synthetase (*purM*; EC: 6.3.3.1), and amidophosphoribosyl transferase (*purF*; EC: 2.4.2.14). A gene encoding carbamate kinase (*arcC*; EC: 2.7.2.2) was not up-regulated by NaP. In the riboflavin metabolic pathway, four genes encoding enzymes and other proteins involved in riboflavin synthesis were significantly up-regulated by NaP, such as riboflavin synthase subunit alpha (*ribE*; EC: 2.5.1.9), riboflavin biosynthesis protein (*ribD*; EC: 3.5.4.26; 1.1.1.193), 6,7-dimethyl-8-ribityllumazine synthase (*ribH*; EC: 2.5.1.78), and riboflavin biosynthesis protein (*ribA*; EC: 3.5.4.25; 4.199.12). In the glycine, serine, and threonine metabolic pathway, five genes encoding enzymes involved in threonine synthesis and down-stream metabolism were up-regulated, including threonine dehydratase (*ilvA*; EC: 4.3.1.19), homoserine kinase (*thrB*; EC: 2.7.1.39), homoserine dehydrogenase (*hom*; EC: 1.1.1.3), threonine synthase (*thrC*; EC: 4.2.3.1), and aspartate kinase (*thrD*; EC: 2.7.2.4). However, three genes were down-regulated in this metabolic pathway, such as phosphoglyceromutase (*gpmI*; EC: 5.4.2.12), glycine cleavage system aminomethyltransferase T (*gcvT*; EC: 2.1.2.10), and glycine betaine aldehyde dehydrogenase (*betB*; EC: 1.2.1.8).

### Function of identified pathways in growth of methicillin-resistant *Staphylococcus aureus*

**Figure 5:**
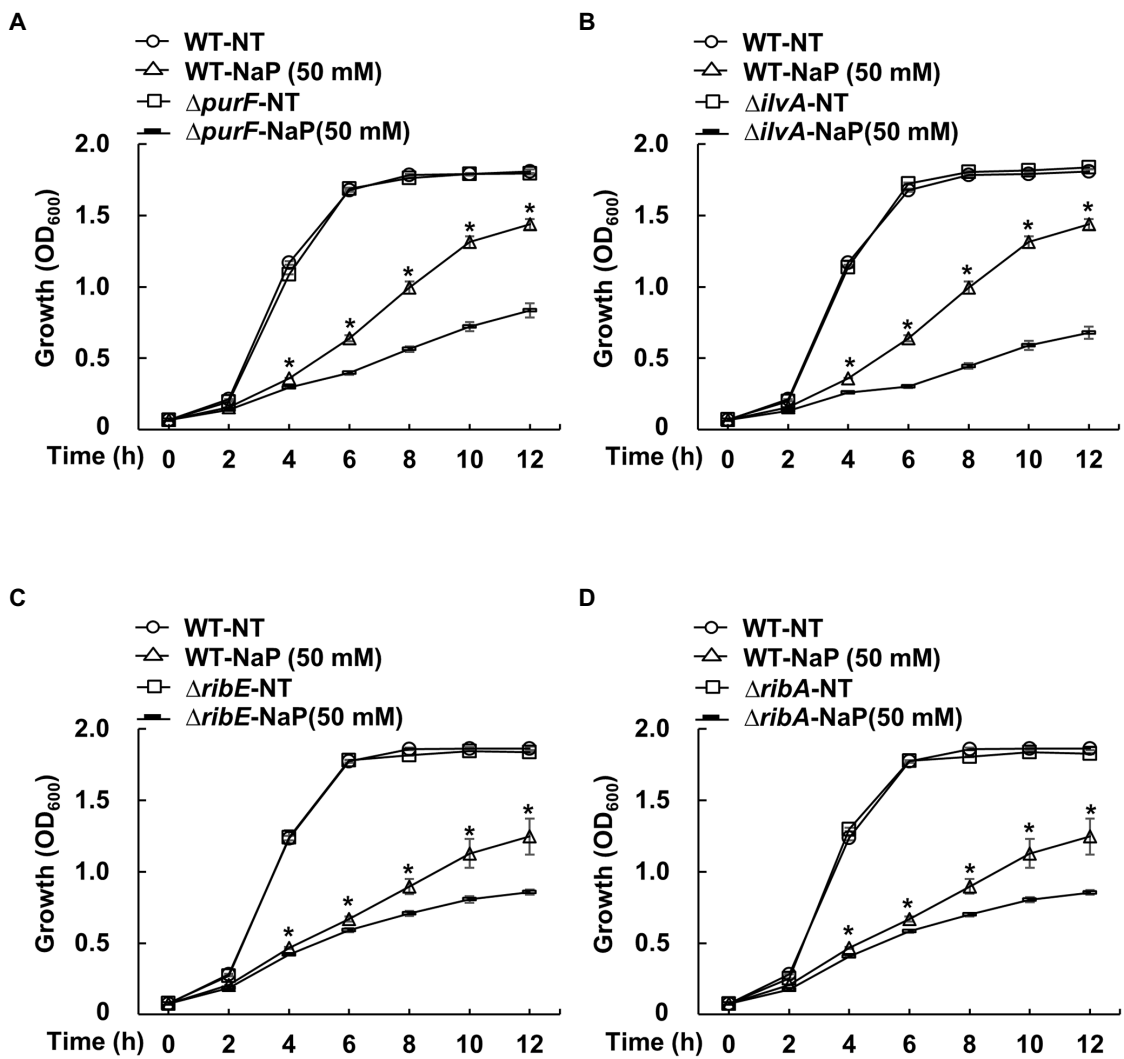
The effect of NaP on growth of MRSA mutant strains deficient for the up-regulated DEGs in the selected metabolic pathways. *S. aureus* USA300 wild-type and mutant strains that lack the up-regulated DEGs of the metabolism pathways including **(A)** *purF* (*∆purF*) for purine metabolism; **(B)** *ilvA* (*∆ilvA*) for glycine, serine, and threonine metabolism; and **(C)** *ribE* (*∆ribE*) and **(D)** *ribA* (*∆ribA*) for riboflavin metabolism were incubated with 50 mM NaP for 12 h under aerobic shaking culture conditions. Bacterial growth was examined by measuring optical density at 600 nm every 2 h up to 12 h. Data shown are the mean ± SEM of triplicate samples. ^*^p < 0.05 compared with the wild-type treated with 50 mM NaP (WT-NaP) at each time point.

To further investigate the role of the selected pathways in the bacteriostatic effect of NaP, bacterial growth of *S. aureus* USA300 strains that lack the up-regulated DEGs in the selected metabolic pathways was evaluated in the presence of NaP and compared with that of wild-type. Among various up-regulated DEGs in the selected pathways, the four DEG-deficient strains *purF* (*∆purF*) for purine metabolism; *ilvA* (*∆ilvA*) for glycine, serine, and threonine metabolism; and *ribE* (*∆ribE*) and *ribA* (*∆ribA*) for riboflavin metabolism were adapted for growth analysis. As shown in Figures 5A–D, all tested mutant strains were more susceptible to NaP compared with wild-type after 4 h. These results suggest that “purine metabolism”; “riboflavin metabolism”; and “glycine, serine, and threonine metabolism” are critical metabolic pathways responsible for the bacteriostatic effect of NaP on MRSA.

## Discussion

MRSA is a serious clinical threat with persistently high morbidity and mortality, suggesting an urgent need for alternative therapeutic strategies. We have previously demonstrated potential utilization of NaP as an effective bacteriostatic agent against MRSA infection (Jeong et al., 2019a). However, the underlying mechanisms were unclear. In the current study, we found genetic evidence for the bacteriostatic effect of NaP on MRSA by transcriptomic analysis using RNA-Seq. In NaP-treated MRSA, 131 and 40 genes were significantly up-regulated and down-regulated, respectively. Moreover, KEGG pathway enrichment analysis showed that “purine metabolism”; “riboflavin metabolism”; and “glycine, serine, and threonine metabolism” were major metabolic pathways altered by NaP. Furthermore, the growth of MRSA strains deficient for *purF*, *ilvA*, *ribE*, or *ribA*, which are up-regulated genes in these metabolic pathways, was more susceptible to NaP compared with wild-type, suggesting that the bacteriostatic effect of NaP on MRSA is mediated through alteration of these pathways. Based on the KEGG pathway enrichment analysis, purine metabolism seems to function as an up-stream metabolic pathway regulating both riboflavin metabolism and glycine, serine, and threonine metabolism (Supplementary Figure S1A). In fact, since purine metabolic pathway produces guanosine triphosphate and glycine, which are essential precursor metabolites for synthesis of riboflavin, and serine and threonine, respectively, it can directly regulate these two metabolic pathways (Averianova et al., 2020; Shimizu and Matsuoka, 2022). Therefore, we concluded that purine metabolism is a central metabolic pathway responsible for NaP-induced bacteriostatic effect in MRSA growth.

In the current study, we found differentially expressed purine metabolic pathway with eight up-regulated DEGs in NaP-treated *S. aureus* USA300. Since nine of 10 up-regulated genes, *purC*, *purS*, *purH*, *purN*, *purD*, *purL*, *purQ*, *purM*, and *purF*, belong to the *pur* purine biosynthetic operon, NaP probably up-regulates purine biosynthesis of MRSA. Similar to our results, enhanced purine synthesis was observed in *E. coli* under stress conditions induced by various antibiotics (Yang et al., 2019). Although purine biosynthesis is critical for bacterial growth *via* DNA and RNA syntheses and ATP energy supply (Zhang et al., 2008), the mechanism by which increased purine biosynthesis during stress conditions is associated with bacterial growth is unclear. Based on recent studies, this can be explained by enhanced DNA damage following increased purine biosynthesis under stress conditions (Yee et al., 2015; Yang et al., 2019; Lopatkin and Yang, 2021). Thus, stress conditions, such as those occurring with antibiotic treatment, induced disruption of the nucleotide pool, causing adenine deficiency and triggering purine biosynthesis. Also, activated central carbon metabolism resulting from increased purine biosynthesis subsequently enhanced reactive oxygen species (ROS) production. Finally, ROS-damaged DNA leads to inhibited growth or increased death of bacteria. Therefore, the bacteriostatic effects of NaP may be affected by up-regulated purine biosynthesis leading to enhanced bacterial DNA damage. This possibility is supported by the higher susceptibility of MRSA mutants involved in purine biosynthesis, such as *purM*, to various stress conditions, including antibiotics, heat, and low pH (Yee et al., 2015).

The riboflavin metabolic pathway was also differentially expressed in MRSA when treated with NaP. In the pathway, the four genes *ribE*, *ribD*, *ribH*, and *ribA* existing in the *rib* riboflavin biosynthetic operon were up-regulated, suggesting that NaP probably enhances MRSA riboflavin biosynthesis. Moreover, since riboflavin was mainly synthesized from guanosine-5′-triphosphate derived from the purine biosynthetic pathway (Garcia-Angulo, 2017), the NaP-induced purine biosynthesis may cause increased riboflavin biosynthesis. The role of NaP-induced riboflavin biosynthesis in the bacteriostatic effect of NaP can be explained. In bacteria, riboflavin plays important roles in bacterial growth through various bacterial metabolic pathways, such as fatty acid metabolism, tricarboxylic acid cycle function, and electron transport chain progression, *via* its derivatives, including flavin mononucleotide and flavin adenine dinucleotide (FAD; Massey, 2000; Lin et al., 2014). However, enhanced riboflavin synthesis can result in excessive ROS production by promoting autoxidation of FAD through FADH2 oxidation, leading to ROS-induced DNA damage (Imlay, 2013). A previous study reported that stress conditions induced by an anti-bacterial reagent in *Edwardsiella tarda* up-regulated riboflavin biosynthesis and subsequent ROS production, leading to ROS-mediated DNA damage and bacterial growth inhibition (Ye et al., 2018). Therefore, excessive ROS production through enhanced riboflavin biosynthesis by NaP may be a potential mechanism responsible for the bacteriostatic effect of NaP on MRSA.

The differentially expressed glycine, serine, and threonine metabolic pathway caused by NaP administration can also inhibit MRSA growth. Up-regulation of four genes involved in threonine biosynthesis from L-aspartate, *hom, thrB, thrC*, and *thrD*, may involve enhanced threonine biosynthesis in MRSA under NaP treatment. Synthesized threonine can then be converted into glycine and serine and, subsequently, pyruvate for energy or into isoleucine (Fang et al., 2021). During the conversion, *ilvA* is responsible for conversion of serine to pyruvate and, together with *sdaAB*, the conversion of threonine into 2-oxobutyrate (2-OBA), a precursor metabolite of isoleucine. However, NaP did not affect the gene expression of enzymes involved in conversion of threonine into glycine or of glycine into serine, suggesting that NaP-induced *ilvA* expression is involved in 2-OBA generation. Of note, 2-OBA is a toxic metabolic intermediate leading to the arrest of cell growth in most microorganisms (LaRossa et al., 1987; Fang et al., 2021). Thus, NaP-induced *ilvA* expression following up-regulated threonine biosynthesis may suppress MRSA growth by excessive 2-OBA production. However, we also observed down-regulation of *gpmI*, g*cvT*, and *betB* in this metabolic pathway; alteration of the *gcvT* gene by NaP is another possible explanation for the inhibitory effect of NaP on MRSA growth. Although glycine is an essential amino acid involved in bacterial growth as a precursor of pyruvate for energy metabolism (Fang et al., 2021), excessive glycine can inhibit growth of various bacteria, including *Bacillus subtilis*, *Streptococcus lactis*, *Streptomyces griseus*, and *S. aureus* (Tezuka and Ohnishi, 2014). To counteract this phenomenon, most bacteria possess a glycine cleavage system as a detoxification mechanism to mediate the oxidative cleavage of glycine into CO_2_, NH_4_^+^, and a methylene group (Kikuchi et al., 2008). Therefore, NaP-induced down-regulation of *gcvT*, which is a key gene of the glycine cleavage system in *S. aureus*, may lead to suppressed MRSA growth by inhibiting this system.

Based on our results, the bacteriostatic effect of NaP against MRSA seems to be mediated by different mechanism compared with other bacterial growth inhibitory substances. According to the previous studies, propionate effectively inhibited bacterial growth of *S. aureus* and *Salmonella typhimurium* by disrupting their intracellular pH homeostasis (Wang et al., 2014; Jacobson et al., 2018). On the other hand, inorganic acid and alkaline shock suppressed *S. aureus* growth by inducing alteration of gene expression for adaptation to pH stress (Anderson et al., 2010). Thus, we have examined the pH changes in the extracellular culture medium of *S. aureus* USA300 in the presence of NaP. However, NaP did not affect pH of the culture medium throughout the experiment (data not shown). Therefore, bacteriostatic effect of NaP might be mediated by altered metabolic pathways, including purine metabolism, riboflavin metabolism, and glycine, serine, and threonine metabolism, but not by pH stress.

Among various antibiotics resistant to *S. aureus*, MRSA is the most prevalent form, with persistently high morbidity and mortality (Turner et al., 2019). Although vancomycin and daptomycin are currently approved anti-bacterials for treatment of MRSA infection (Liu et al., 2011), reports on treatment failure with vancomycin and decreased susceptibility to daptomycin necessitate the development of alternative strategies (Ortwine and Bhavan, 2018). Recently, semisynthetic lipoglycopeptides, including telavancin, dalbavancin and oritavancin, have been suggested as alternative anti-bacterials with reliable bactericidal activity against MRSA. However, these often accompany various adverse effects such as hypersensitivity, anaphylaxis, and osteomyelitis (Ortwine and Bhavan, 2018). In our previous study, bacterial load and dermonecrosis in the co-treatment group of *S. aureus* USA300 and 50 mM NaP were effectively reduced compared to *S. aureus* USA300 single-treatment group in a murine MRSA skin infection model (Jeong et al., 2019a). Furthermore, the same concentration of NaP (50 mM) did not induce pathology or inflammation in the murine MRSA skin infection model, suggesting a non-toxic effect of NaP at 50 mM. In addition, the non-toxic effect of high concentration of NaP even at 200 mM has been demonstrated in a mouse with a month of NaP oral administration (Ciarlo et al., 2016). Moreover, since propionate is present in various sites of the human body, such as liver, lung, and colon, its utilization as a bacteriostatic agent against MRSA suggests biocompatibility. The clinical applications of propionate as a potential antimicrobial agent would be appropriate as a bacteriostatic agent for the prevention of MRSA infection and/or its disease progression rather than a bactericidal agent for MRSA treatment. Since the underlying mechanisms responsible for the bacteriostatic effect of propionate have remained unclear so far, the current findings might contribute to the development of an effective and safe antimicrobial agent using propionate against MRSA infection.

## Data availability statement

The raw data associated with this study are accessible through the NCBI Sequence Read Archive (SRA) database under BioProject ID PRJNA887926.

## Author contributions

JI, DL, O-JP, and SH designed the research. JI, DL, and, SN carried out the experiments. JI, DL, O-JP, SN, JP, C-HY, and SH analyzed and interpreted the data. JI, DL, O-JP, SN, JP, C-HY, and SH prepared and reviewed the manuscript. All authors contributed to the article and approved the submitted version.

## Funding

This work was supported by the National Research Foundation of Korea (NRF) funded by the Ministry of Science and ICT (NRF-2018R1A5A2024418, NRF-2019R1A2C2007041, NRF-2022M3A9F3082330, and RS-2022-00164722).

## Conflict of interest

SN and JP were employed by 3BIGS Co., Ltd.

The remaining authors declare that the research was conducted in the absence of any commercial or financial relationships that could be construed as a potential conflict of interest.

## Publisher’s note

All claims expressed in this article are solely those of the authors and do not necessarily represent those of their affiliated organizations, or those of the publisher, the editors and the reviewers. Any product that may be evaluated in this article, or claim that may be made by its manufacturer, is not guaranteed or endorsed by the publisher.

## Supplementary material

The Supplementary material for this article can be found online at: https://www.frontiersin.org/articles/10.3389/fmicb.2022.1063650/full#supplementary-material

## Footnotes

^1^ https://www.bioinformatics.babraham.ac.uk/projects/fastqc
