# Supplementary material for: RNA-Seq-based transcriptome analysis of methicillin-resistant Staphylococcus aureus growth inhibition by propionate
Source: Front Microbiol. 2022 Dec 22;13:1063650. doi: 10.3389/fmicb.2022.1063650 (PMC9814166; doi:10.3389/fmicb.2022.1063650)
Supplement: Supplementary file 1 [file Table_1.DOCX]

**SUPPLEMENTARY TABLE 1 |** Sequences of primers used for real-time PCR analysis.

| Target gene | Primer orientation | Primer sequence (5’-3’) |
| --- | --- | --- |
| *adh* | Forward | GTCGTGAAACACTTTGCCGT |
|  | Reverse | ACGCTGCTGCTGGATCTAAT |
| *purN* | Forward | CTAGCTGGCTACATGCGTCT |
|  | Reverse | TTGGCCTATTGCGTCAATCC |
| *purF* | Forward | CGTGAGCAAGGTGTGAGAGT |
|  | Reverse | CGAATTGTCGTACCGCGAAC |
| *purM* | Forward | ACGACAGGTGCAGAACCATT |
|  | Reverse | TTTCAGCAGTCTCTCCACCG |
| *tpiA* | Forward | ACGAAGAGCGTGAAAGTGGT |
|  | Reverse | TACCAGTTCCGATTGCCCAG |
| *pgm* | Forward | AGAGCAGCGCAATTATCGGA |
|  | Reverse | TTTCGAAGACGATAGCCGCA |
| *gap* | Forward | GCAGCGGCAGAAAACATCAT |
|  | Reverse | ACGTTGTGCACCACCATCTA |
| *pgk* | Forward | GCGCACACTGTTGTATGGAA |
|  | Reverse | CACCGCCACCGATAATCGTA |
| 16s rRNA | Forward | AAGCAACGCGAAGAACCTTA |
|  | Reverse | GTCTCGCTAGAGTGCCCAAC |
