# Supplementary material for: RNA-Seq-based transcriptome analysis of methicillin-resistant Staphylococcus aureus growth inhibition by propionate
Source: Front Microbiol. 2022 Dec 22;13:1063650. doi: 10.3389/fmicb.2022.1063650 (PMC9814166; doi:10.3389/fmicb.2022.1063650)
Supplement: Supplementary file 2 [file Table_2.DOCX]

**SUPPLEMENTARY TABLE 2 |** Summary of up-regulated DEGs by NaP treatment.

| Locus Tag | Gene | Log_2_ FC | P-value | Description |
| --- | --- | --- | --- | --- |
| SAUSA300_RS09605 | *splD* | 6.95 | 2.59×10^-205^ | Serine protease SplD |
| SAUSA300_RS09600 | *splE* | 6.68 | 5.50×10^-161^ | Serine protease SplE |
| SAUSA300_RS09610 | *splC* | 6.58 | 1.28×10^-156^ | Serine protease SplC |
| SAUSA300_RS09620 | *splA* | 6.57 | 3.94×10^-188^ | Serine protease SplA |
| SAUSA300_RS09595 | *splF* | 6.52 | 4.37×10^-149^ | Serine protease SplF |
| SAUSA300_RS09615 | *splB* | 6.45 | 1.45×10^-92^ | Serine protease SplB |
| SAUSA300_RS08885 | *thrS* | 5.61 | 1.76×10^-300^ | Threonyl-tRNA synthetase |
| SAUSA300_RS05680 | *flr* | 5.15 | 2.24×10^-232^ | Formyl peptide receptor-like 1 inhibitory protein |
| SAUSA300_RS07250 | *ilvA* | 4.93 | 8.37×10^-170^ | Threonine dehydratase |
| SAUSA300_RS01370 | *lrgB* | 4.77 | 6.41×10^-187^ | Antiholin-like protein LrgB |
| SAUSA300_RS01365 | *lrgA* | 4.70 | 8.33×10^-81^ | Murein hydrolase regulator LrgA |
| SAUSA300_RS10530 | *chs* | 4.62 | 2.45×10^-46^ | Chemotaxis-inhibiting protein CHIPS |
| SAUSA300_RS09370 | *ribD* | 4.50 | 1.59×10^-134^ | Riboflavin biosynthesis protein |
| SAUSA300_RS09360 | *ribA* | 4.46 | 8.87×10^-177^ | Riboflavin biosynthesis protein |
| SAUSA300_RS07245 | *-* | 4.43 | 1.23×10^-121^ | Amino acid permease |
| SAUSA300_RS09365 | *ribE* | 4.41 | 5.91×10^-168^ | Riboflavin synthase subunit alpha |
| SAUSA300_RS09355 | *ribH* | 4.40 | 5.16×10^-104^ | 6,7-dimethyl-8-ribityllumazine synthase |
| SAUSA300_RS10850 | *lukH* | 4.26 | 2.80×10^-178^ | Succinyl-diaminopimelate desuccinylase |
| SAUSA300_RS10845 | *lukG* | 4.20 | 4.41×10^-99^ | Gamma-hemolysin subunit B |
| SAUSA300_RS13060 | *sbi* | 4.16 | 6.40×10^-242^ | IgG-binding protein SBI |
| SAUSA300_RS04395 | *ear* | 4.11 | 3.72×10^-147^ | Ear protein |
| SAUSA300_RS01130 | *-* | 3.94 | 5.95×10^-9^ | Membrane protein |
| SAUSA300_RS07240 | *norB* | 3.83 | 1.08×10^-213^ | Quinolone resistance protein NorB |
| SAUSA300_RS07255 | *ald* | 3.72 | 5.02×10^-144^ | Alanine dehydrogenase |
| SAUSA300_RS06655 | *thrB* | 3.72 | 3.22×10^-196^ | Homoserine kinase |
| SAUSA300_RS10505 | *hlb-1* | 3.71 | 3.18×10^-42^ | Hypothetical protein |
| SAUSA300_RS04185 | *nuc* | 3.64 | 2.37×10^-47^ | Thermonuclease |
| SAUSA300_RS02675 | *rrsB* | 3.60 | 5.30×10^-8^ | 16S ribosomal RNA |
| SAUSA300_RS06645 | *hom* | 3.57 | 2.84×10^-101^ | Homoserine dehydrogenase |
| SAUSA300_RS00515 | *plc* | 3.56 | 1.66×10^-125^ | 1-phosphatidylinositol phosphodiesterase |
| SAUSA300_RS06650 | *thrC* | 3.56 | 1.92×10^-172^ | Threonine synthase |
| SAUSA300_RS13075 | *hlgC* | 3.50 | 5.61×10^-74^ | Gamma-hemolysin component C |
| SAUSA300_RS03720 | *saeP* | 3.36 | 1.42×10^-159^ | Hypothetical protein |
| SAUSA300_RS13080 | *hlgB* | 3.35 | 2.48×10^-127^ | Gamma-hemolysin component B |
| SAUSA300_RS09475 | *tnp2* | 3.30 | 2.52×10^-48^ | IS200/IS605 family transposase |
| SAUSA300_RS13765 | *-* | 3.20 | 1.03×10^-27^ | Hypothetical protein |
| SAUSA300_RS10340 | *scpA* | 3.17 | 9.91×10^-80^ | Staphopain A |
| SAUSA300_RS02700 | *pdxS* | 3.08 | 4.09×10^-143^ | Pyridoxal biosynthesis lyase PdxS |
| SAUSA300_RS07535 | *-* | 3.06 | 3.84×10^-7^ | Hypothetical protein |
| SAUSA300_RS02705 | *pdxT* | 2.99 | 2.77×10^-49^ | Pyridoxal 5-phosphate synthase glutaminase subunit PdxT |
| SAUSA300_RS14375 | *asp3* | 2.90 | 1.09×10^-54^ | Accessory Sec system protein Asp3 |
| SAUSA300_RS10525 | *scn* | 2.89 | 3.74×10^-117^ | Hypothetical protein |
| SAUSA300_RS09630 | *-* | 2.88 | 1.69×10^-19^ | Hypothetical protein |
| SAUSA300_RS05690 | *efb* | 2.86 | 1.03×10^-30^ | Fibrinogen-binding protein |
| SAUSA300_RS03715 | *saeQ* | 2.82 | 8.92×10^-86^ | Hypothetical protein |
| SAUSA300_RS06640 | *thrD* | 2.82 | 2.58×10^-54^ | Aspartate kinase |
| SAUSA300_RS10010 | *trnaG* | 2.80 | 1.12×10^-13^ | tRNA-Gly |
| SAUSA300_RS10005 | *trnaL* | 2.76 | 1.14×10^-4^ | tRNA-Leu |
| SAUSA300_RS01635 | *-* | 2.76 | 1.86×10^-16^ | 5'-nucleotidase |
| SAUSA300_RS06045 | *rpmB* | 2.74 | 4.03×10^-29^ | 50S ribosomal protein L28 |
| SAUSA300_RS00930 | *-* | 2.73 | 1.11×10^-92^ | Acyl-CoA dehydrogenase |
| SAUSA300_RS04655 | *glpQ* | 2.73 | 5.79×10^-55^ | Glycerophosphoryl diester phosphodiesterase |
| SAUSA300_RS14395 | *sasA* | 2.72 | 4.22×10^-78^ | Serine-rich repeat glycoprotein adhesion SasA |
| SAUSA300_RS14380 | *asp2* | 2.72 | 1.09×10^-81^ | Accessory Sec system protein Asp2 |
| SAUSA300_RS13330 | *cntF* | 2.68 | 1.36×10^-43^ | ABC transporter ATP-binding protein |
| SAUSA300_RS03710 | *saeR* | 2.66 | 1.18×10^-32^ | DNA-binding response regulator SaeR |
| SAUSA300_RS10015 | *trnaL* | 2.66 | 5.13×10^-14^ | tRNA-Leu |
| SAUSA300_RS05670 | *ecb* | 2.65 | 4.52×10^-29^ | Fibrinogen-binding protein |
| SAUSA300_RS10020 | *trnaK* | 2.64 | 5.01×10^-6^ | tRNA-Lys |
| SAUSA300_RS10030 | *tranV* | 2.63 | 2.06×10^-4^ | tRNA-Val |
| SAUSA300_RS07540 | *lukF-PV* | 2.60 | 7.32×10^-47^ | Panton-Valentine leukocidin, LukF-PV |
| SAUSA300_RS04180 | *-* | 2.60 | 4.52×10^-74^ | Hypothetical protein |
| SAUSA300_RS05695 | *scc* | 2.57 | 6.10×10^-17^ | Fibrinogen-binding protein |
| SAUSA300_RS00710 | *sasD* | 2.56 | 1.82×10^-101^ | Cell wall surface anchor family protein |
| SAUSA300_RS07545 | *lukS-PV* | 2.55 | 6.59×10^-41^ | Panton-Valentine leukocidin, LukS-PV |
| SAUSA300_RS11685 | *rrfE* | 2.55 | 3.19×10^-2^ | 5S ribosomal RNA |
| SAUSA300_RS02035 | *tcyP* | 2.54 | 1.10×10^-94^ | L-cystine transporter |
| SAUSA300_RS03705 | *saeS* | 2.54 | 2.51×10^-32^ | Sensor histidine kinase SaeS |
| SAUSA300_RS02810 | *rplL* | 2.53 | 8.61×10^-81^ | 50S ribosomal protein L7/L12 |
| SAUSA300_RS10000 | *trnaR* | 2.53 | 4.56×10^-5^ | tRNA-Arg |
| SAUSA300_RS05245 | *purD* | 2.51 | 4.00×10^-83^ | Phosphoribosylamine--glycine ligase |
| SAUSA300_RS09980 | *trnaM* | 2.50 | 4.60×10^-5^ | tRNA-Met |
| SAUSA300_RS05205 | *purC* | 2.50 | 1.70×10^-30^ | phosphoribosylaminoimidazole-succinocarboxamide synthase |
| SAUSA300_RS01755 | *ulaA* | 2.50 | 1.45×10^-38^ | PTS system ascorbate-specific transporter subunit IIC |
| SAUSA300_RS12440 | *ssaA* | 2.48 | 8.03×10^-26^ | Secretory antigen precursor SsaA |
| SAUSA300_RS05230 | *purM* | 2.46 | 1.70×10^-113^ | Phosphoribosylaminoimidazole synthetase |
| SAUSA300_RS05760 | *arcC* | 2.44 | 2.95×10^-55^ | Carbamate kinase |
| SAUSA300_RS02670 | *trnaA* | 2.44 | 4.46×10^-4^ | tRNA-Ala |
| SAUSA300_RS01025 | *murR* | 2.44 | 2.87×10^-27^ | MurR/RpiR family transcriptional regulator |
| SAUSA300_RS06660 | *-* | 2.43 | 1.38×10^-40^ | HAD superfamily hydrolase |
| SAUSA300_RS10025 | *trnaT* | 2.43 | 3.24×10^-10^ | tRNA-Thr |
| SAUSA300_RS02665 | *rrfG* | 2.42 | 7.62×10^-3^ | 5S ribosomal RNA |
| SAUSA300_RS09995 | *trnaP* | 2.41 | 1.28×10^-10^ | tRNA-Pro |
| SAUSA300_RS01760 | *-* | 2.40 | 6.13×10^-27^ | PTS lactose transporter subunit IIB |
| SAUSA300_RS02805 | *rplJ* | 2.39 | 2.47×10^-49^ | 50S ribosomal protein L10 |
| SAUSA300_RS13605 | *-* | 2.38 | 4.65×10^-48^ | ABC transporter ATP-binding protein |
| SAUSA300_RS13070 | *hlgA* | 2.36 | 5.71×10^-30^ | Gamma-hemolysin component A |
| SAUSA300_RS09975 | *trnaS* | 2.32 | 9.26×10^-10^ | tRNA-Ser |
| SAUSA300_RS09985 | *trnaM* | 2.32 | 6.02×10^-4^ | tRNA-Met |
| SAUSA300_RS05235 | *purN* | 2.31 | 1.80×10^-23^ | Phosphoribosylglycinamide formyltransferase |
| SAUSA300_RS05225 | *purF* | 2.31 | 9.40×10^-85^ | Amidophosphoribosyltransferase |
| SAUSA300_RS05385 | *potB* | 2.29 | 2.30×10^-38^ | Spermidine/putrescine ABC transporter permease |
| SAUSA300_RS00955 | *-* | 2.28 | 1.01×10^-29^ | 4'-phosphopantetheinyl transferase superfamily protein |
| SAUSA300_RS05240 | *purH* | 2.28 | 1.25×10^-26^ | Bifunctional phosphoribosylaminoimidazolecarboxamide formyltransferase/IMP cyclohydrolase |
| SAUSA300_RS09990 | *trnaA* | 2.26 | 4.58×10^-4^ | tRNA-Ala |
| SAUSA300_RS12730 | *tcaR* | 2.24 | 2.43×10^-34^ | Transcriptional regulator TcaR |
| SAUSA300_RS09930 | *trnaH* | 2.23 | 3.77×10^-3^ | tRNA-His |
| SAUSA300_RS05220 | *purL* | 2.22 | 1.41×10^-99^ | Phosphoribosylformylglycinamidine synthase II |
| SAUSA300_RS13610 | *-* | 2.17 | 7.85×10^-23^ | ABC tranporter permease |
| SAUSA300_RS01125 | *-* | 2.17 | 3.17×10^-41^ | Xylose isomerase |
| SAUSA300_RS11675 | *trnaE* | 2.16 | 2.34×10^-8^ | tRNA-Glu |
| SAUSA300_RS01020 | *murP* | 2.15 | 1.26×10^-50^ | Sucrose-specific PTS transporter protein |
| SAUSA300_RS10050 | *-* | 2.15 | 7.54×10^-6^ | tRNA-Ile |
| SAUSA300_RS14385 | *asp1* | 2.15 | 8.85×10^-35^ | Accessory Sec system protein Asp1 |
| SAUSA300_RS12070 | *rplR* | 2.15 | 1.66×10^-33^ | 50S ribosomal protein L18 |
| SAUSA300_RS01015 | *murQ* | 2.14 | 2.16×10^-73^ | N-acetylmuramic acid-6-phosphate etherase |
| SAUSA300_RS14285 | *arcD* | 2.14 | 2.68×10^-27^ | Arginine/ornithine antiporter |
| SAUSA300_RS05390 | *potC* | 2.13 | 7.52×10^-39^ | Spermidine/putrescine ABC transporter permease |
| SAUSA300_RS05215 | *purQ* | 2.13 | 9.22×10^-48^ | Phosphoribosylformylglycinamidine synthase I |
| SAUSA300_RS12075 | *rplF* | 2.13 | 1.15×10^-47^ | 50S ribosomal protein L6 |
| SAUSA300_RS11680 | *trnaA* | 2.12 | 6.29×10^-8^ | tRNA-Asn |
| SAUSA300_RS13270 | *pnbA* | 2.11 | 2.03×10^-77^ | Para-nitrobenzyl esterase |
| SAUSA300_RS05210 | *purS* | 2.11 | 6.41×10^-17^ | Phosphoribosylformylglycinamidine synthase |
| SAUSA300_RS01765 | *-* | 2.10 | 4.57×10^-18^ | PTS ascorbate transporter subunit IIA |
| SAUSA300_RS01160 | *pflA* | 2.10 | 1.74×10^-35^ | Pyruvate formate-lyase activating enzyme |
| SAUSA300_RS12065 | *rpsE* | 2.09 | 1.98×10^-35^ | 30S ribosomal protein S5 |
| SAUSA300_RS12060 | *rpmD* | 2.09 | 1.13×10^-34^ | 50S ribosomal protein L30 |
| SAUSA300_RS09935 | *trnaW* | 2.09 | 4.0×10^-4^ | tRNA-Trp |
| SAUSA300_RS01010 | *mupG* | 2.09 | 1.39×10^-47^ | Hypothetical protein |
| SAUSA300_RS01840 | *tatC* | 2.07 | 4.63×10^-27^ | Sec-independent protein translocase TatC |
| SAUSA300_RS01155 | *pflB* | 2.06 | 6.45×10^-48^ | Formate acetyltransferase |
| SAUSA300_RS02440 | *rrsA* | 2.06 | 1.67×10^-3^ | 16S ribosomal RNA |
| SAUSA300_RS12700 | *-* | 2.06 | 2.14×10^-24^ | HlyD family secretion protein |
| SAUSA300_RS00585 | *spa* | 2.05 | 1.31×10^-80^ | Peptidoglycan-binding protein LysM |
| SAUSA300_RS14310 | *aur* | 2.04 | 5.83×10^-23^ | Zinc metalloproteinase aureolysin |
| SAUSA300_RS10550 | *-* | 2.03 | 8.60×10^-5^ | Holin |
| SAUSA300_RS11100 | *trnaG* | 2.02 | 2.77×10^-2^ | tRNA-Gly |
| SAUSA300_RS03190 | *adh* | 2.01 | 2.23×10^-28^ | Alcohol dehydrogenase |
| SAUSA300_RS02680 | *trnaI* | 2.00 | 8.44×10^-3^ | tRNA-Ile |
| SAUSA300_RS13425 | *-* | 2.00 | 8.66×10^-39^ | Hypothetical protein |
| SAUSA300_RS02575 | *rplY* | 2.00 | 5.84×10^-34^ | 50S ribosomal protein L25/general stress protein Ctc |
