# Supplementary material for: RNA-Seq-based transcriptome analysis of methicillin-resistant Staphylococcus aureus growth inhibition by propionate
Source: Front Microbiol. 2022 Dec 22;13:1063650. doi: 10.3389/fmicb.2022.1063650 (PMC9814166; doi:10.3389/fmicb.2022.1063650)
Supplement: Supplementary file 3 [file Table_3.DOCX]

**SUPPLEMENTARY TABLE 3 |** Summary of down-regulated DEGs by NaP treatment.

| Locus Tag | Gene | Log_2_ FC | P-value | Description |
| --- | --- | --- | --- | --- |
| SAUSA300_RS12750 | *hrtB* | -6.52 | 3.12×10^-130^ | ABC transporter permease |
| SAUSA300_RS12745 | *hrtA* | -5.48 | 5.37×10^-162^ | ABC transporter ATP-binding protein |
| SAUSA300_RS07735 | *-* | -3.97 | 4.85×10^-2^ | PhiSLT ORF 82-like protein |
| SAUSA300_RS07530 | *-* | -3.81 | 6.30×10^-3^ | Putative lipoprotein |
| SAUSA300_RS03675 | *fruA* | -3.70 | 4.03×10^-184^ | Fructose specific permease |
| SAUSA300_RS03670 | *fruB* | -3.41 | 7.46×10^-77^ | Fructose 1-phosphate kinase |
| SAUSA300_RS10475 | *pmtR* | -3.25 | 1.32×10^-96^ | GntR family transcriptional regulator |
| SAUSA300_RS10460 | *pmtC* | -3.25 | 7.89×10^-85^ | ABC transporter ATP-binding protein |
| SAUSA300_RS04075 | *gapR* | -3.24 | 2.71×10^-52^ | Glycolytic operon regulator |
| SAUSA300_RS10465 | *pmtB* | -3.20 | 1.50×10^-14^ | ABC-2 transporter family protein |
| SAUSA300_RS10470 | *pmtA* | -3.17 | 1.94×10^-69^ | ABC transporter ATP-binding protein |
| SAUSA300_RS10455 | *pmtD* | -2.87 | 3.06×10^-19^ | Membrane protein |
| SAUSA300_RS14135 | *betB* | -2.85 | 1.63×10^-66^ | Glycine betaine aldehyde dehydrogenase |
| SAUSA300_RS12780 | *lctP2* | -2.80 | 1.97×10^-120^ | L-lactate permease |
| SAUSA300_RS11565 | *-* | -2.68 | 1.70×10^-68^ | Lytic regulatory protein |
| SAUSA300_RS03665 | *fruR* | -2.62 | 2.54×10^-23^ | DeoR family transcriptional regulator |
| SAUSA300_RS04085 | *pgk* | -2.57 | 2.59×10^-76^ | Phosphoglycerate kinase |
| SAUSA300_RS04080 | *gap* | -2.49 | 1.66×10^-63^ | Glyceraldehyde-3-phosphate dehydrogenase, type I |
| SAUSA300_RS15260 | *-* | -2.46 | 6.38×10^-47^ | Hypothetical protein |
| SAUSA300_RS02025 | *ahpC* | -2.43 | 2.63×10^-66^ | Alkyl hydroperoxide reductase subunit C |
| SAUSA300_RS04535 | *nfu* | -2.43 | 7.34×10^-31^ | NifU family protein |
| SAUSA300_RS14030 | *-* | -2.40 | 9.17×10^-37^ | VOC family protein |
| SAUSA300_RS01470 | *-* | -2.33 | 1.26×10^-29^ | Hypothetical protein |
| SAUSA300_RS04090 | *tpiA* | -2.28 | 7.51×10^-50^ | Triosephosphate isomerase |
| SAUSA300_RS09775 | *-* | -2.26 | 7.95×10^-82^ | Putative ABC transporter protein EcsB |
| SAUSA300_RS13555 | *gntR* | -2.23 | 2.00×10^-18^ | Gluconate operon transcriptional repressor |
| SAUSA300_RS13585 | *-* | -2.22 | 1.01×10^-50^ | DedA family protein |
| SAUSA300_RS04095 | *gpmI* | -2.16 | 1.05×10^-53^ | Phosphoglyceromutase |
| SAUSA300_RS06270 | *rimP* | -2.14 | 2.14×10^-49^ | Ribosome maturation factor |
| SAUSA300_RS06445 | *glpD* | -2.14 | 3.50×10^-43^ | Aerobic glycerol-3-phosphate dehydrogenase |
| SAUSA300_RS00125 | *walJ* | -2.10 | 5.82×10^-24^ | Metallo-beta-lactamase fold metallo-hydrolase |
| SAUSA300_RS13655 | *frp* | -2.08 | 7.64×10^-56^ | NAD(P)H-flavin oxidoreductase |
| SAUSA300_RS06255 | *rasP* | -2.08 | 3.05×10^-50^ | RIP metalloprotease RseP |
| SAUSA300_RS05000 | *-* | -2.07 | 8.11×10^-31^ | DUF2187 domain-containing protein |
| SAUSA300_RS13240 | *opuCb* | -2.07 | 1.62×10^-70^ | Glycine betaine/carnitine/choline ABC transporter |
| SAUSA300_RS08175 | *gcvT* | -2.06 | 7.07×10^-34^ | Glycine cleavage system aminomethyltransferase T |
| SAUSA300_RS08020 | *argR* | -2.05 | 8.10×10^-11^ | Arginine repressor |
| SAUSA300_RS03215 | *-* | -2.03 | 1.58×10^-43^ | Putative iron compound ABC transporter iron compound-binding protein |
| SAUSA300_RS12770 | *-* | -2.01 | 4.65×10^-29^ | DEF3021 domain-containing protein |
| SAUSA300_RS11465 | *-* | -2.01 | 2.36×10^-23^ | Acetyltransferase |
