# Supplementary material for: RNA-Seq-based transcriptome analysis of methicillin-resistant Staphylococcus aureus growth inhibition by propionate
Source: Front Microbiol. 2022 Dec 22;13:1063650. doi: 10.3389/fmicb.2022.1063650 (PMC9814166; doi:10.3389/fmicb.2022.1063650)
Supplement: Supplementary file 4 [file Table_4.DOCX]

**SUPPLEMENTARY TABLE 4 |** GO enrichment analysis of DEGs by NaP treatment.

| Term | | GO ID | No. of genes | Gene name |
| --- | --- | --- | --- | --- |
| Biological process | Localization | GO:0051179 | Up (8) | *asp3; asp2; asp1; potB; potC; murP; ulaA ; SAUSA300_RS01760* |
|  |  |  | Down (3) | *opuCb; pmtD; fruD* |
|  | Cellular process | GO:0009987 | Up (39) | *lrgA; lrgB; lukG; lukH; hlgA; hlgB; hlgC; lukS-PV; saeR; hom; thrD; thrB; thrC; ilvA; arcD; pdxT; ald; arcC; purF; purQ; thrS; pdxS; purC; purD; purH; purL; purM; purN; purS; rplY; rplF; rplJ; rplL; rplR; rpmB; rpmD; rpsE; ribA; ribH* |
|  |  |  | Down (9) | *argR; nfu; gpmI; pgk; tpiA; gcvT; betB; fruB; glpD* |
|  | Component organization/biogenesis | GO:0071840 | Up (1) | *rplJ* |
|  |  |  | Down (3) | *argR; nfu; rimP* |
|  | Biological regulation | GO:0065007 | Up (1) | *saeR* |
|  |  |  | Down (2) | *betB; gcvT* |
|  | Multi-organism process | GO:0051704 | Up (15) | *spa; flr; scpA; scn; lukG; lukH; sasA; chs; hlgA; hlgB; hlgC; lukS-PV; saeR; saeS; sbi;* |
|  |  |  | Down (0) | *-* |
|  | Response to stimulus | GO:0050896 | Up (2) | *saeR; norB* |
|  |  |  | Down (1) | *ahpC* |
|  | Biological adhesion | GO:0022610 | Up (1) | *sasA* |
|  |  |  | Down (0) | *-* |
|  | Signaling | GO:0023052 | Up (1) | *saeR* |
|  |  |  | Down (0) | *-* |
|  | Regulation of biological process | GO:0050789 | Up (1) | *saeR* |
|  |  |  | Down (0) |  |
|  | Cell killing | GO:0001906 | Up (6) | *lukG; lukH; hlgA; hlgB; hlgC; lukS-PV* |
|  |  |  | Down (0) | *-* |
| Molecular function | Catalytic activity | GO:0003824 | Up (43) | *saeS; splD; splA; splB; splC; splE; scpA; adh; hom; ald; pflA; thrC; plc; pdxS; pdxT; ilvA; ribA; pflB; purF; thrB; murP; arc; thrD; purN; purH; ribH; ribE; thrS; purD; purC; purM; purL; purQ; purS; pnbA; nuc; glpQ; cntF*  *SAUSA300_RS00930; SAUSA300_RS00955;*  *SAUSA300_RS01760;*  *SAUSA300_RS06660;*  *SAUSA300_RS13605* |
|  |  |  | Down (17) | *frp; ahpC; glpD; betB; gap; gcvT; fruB; fruA; pgk; walJ; opuCb; pmtC; pmtA; hrtA; gpmI; tpiA; SAUSA300_RS11465* |
|  | Transporter activity | GO:0005215 | Up (6) | *norB; murP; arcD; tcyP; SAUSA300_RS07245; SAUSA300_RS01760;* |
|  |  |  | Down (2) | *fruA: lctP2* |
|  | Binding | GO:0005488 | Up (35) | *ecb; efb; nuc; saeR; rplF; rplR; rpsE; rplY; rplJ; thrS; ilvA; hom; ribA; thrD; cntF; arcC; purC; purD; purL; purM; purQ; purS; saeS; thrB; thrC; murR; pflA; purF; adh; sasA; spa; sbi;*  *SAUSA300_RS00930;*  *SAUSA300_RS13605;*  *SAUSA300_RS00955* |
|  |  |  | Down (12) | *argR; gntR; gap; pmtC; pmtA; hrtA; fruB; pgk; nfu; betB; gpmI; gapR* |
|  | Antioxidant activity | GO:0016209 | Up (0) | *-* |
|  |  |  | Down (1) | *ahpC* |
|  | Structural molecule activity | GO:0005198 | Up (8) | *rplY; rplF; rplJ; rplL; rplR; rpmB; rpmD; rpsE* |
|  |  |  | Down (0) | *-* |
|  | Transcription regulator activity | GO:0140110 | Up (2) | *murR; tcaR* |
|  |  |  | Down (3) | *pmtR; argR; gntR* |
| Cellular component | Cell part | GO:0044464 | Up (34) | *spa; sasD; sasA; rplY; rplF; rplL; rplR; rpmB; rpsE; rplJ; rpmD; thrD; arcC; pflA; pflB; purL; purM; purQ; purS; saeR; thrB; thrS; murP; lrgA; lrgB; norB; potB; potC; saeS; sbi; ulaA; arcD; ribH; SAUSA300_RS01635* |
|  |  |  | Down (13) | *glpD; rimP; ahpC; argR; gpmI; pgk; tpiA; pmtD; hrtA; hrtB; fruA; opuCb; lctP2* |
|  | Cell | GO:0005623 | Up (34) | *spa; sasD; sasA; rplY; rplF; rplL; rplR; rpmB; rpsE; rplJ; rpmD; thrD; arcC; pflA; pflB; purL; purM; purQ; purS; saeR; thrB; thrS; murP; lrgA; lrgB; norB; potB; potC; saeS; sbi; ulaA; arcD; ribH; SAUSA300_RS01635* |
|  |  |  | Down (13) | *glpD; rimP; ahpC; argR; gpmI; pgk; tpiA; pmtD: hrtA; hrtB; fruA; opuCb; lctP2* |
|  | Protein-containing complex | GO:0032991 | Up (9) | *ribH; rpsE; rplJ; rpmD; rplY; rplF; rplL; rplR; rpmB* |
|  |  |  | Down (1) | *glpD* |
|  | Membrane | GO:0016020 | Up (20) | *spa; sasA; sasD; murP; lrgA; lrgB; tcyP; saeQ; norB; nuc; potB; potC; saeS; ulaA; arcD; sbi; SAUSA300_RS07245;*  *SAUSA300_RS12700;*  *SAUSA300_RS13610;*  *SAUSA300_RS01635* |
|  |  |  | Down (13) | *pmtD; pmtB; hrtB; fruA; opuCb; lctP2; hrtA; SAUSA300_RS01470; SAUSA300_RS15260; SAUSA300_RS09775; SAUSA300_RS11565; SAUSA300_RS12770; SAUSA300_RS13585* |
|  | Extracellular region | GO:0005576 | Up (22) | *spa; sasD; flr; scc; scpA; scn; lukG; lukH; sasA; chs; hlgA; hlgB; hlgC; lukS-PV; sbi; splA; splB; splC; splD; splE; ecb; efb;* |
|  |  |  | Down (0) | *-* |
|  | Organelle | GO:0043226 | Up (8) | *rplY; rplF; rplL; rplR; rpmB; rpsE; rplJ; rpmD* |
|  |  |  | Down (0) | *-* |
|  | Membrane part | GO:0044425 | Up (16) | *sasD; murP; lrgA; lrgB; tcyP; saeQ; norB; nuc; potB; potC; saeS; ulaA; arcD; SAUSA300_RS07245; SAUSA300_RS12700; SAUSA300_RS13610* |
|  |  |  | Down (12) | *pmtD ; pmtB; hrtB; fruA; opuCb; lctP2; SAUSA300_RS01470; SAUSA300_RS15260; SAUSA300_RS09775; SAUSA300_RS11565; SAUSA300_RS12770; SAUSA300_RS13585* |
|  | Extracellular region part | GO:0044421 | Up (2) | *ecb; efb* |
|  |  |  | Down (0) | *-* |
|  | Organelle part | GO:0044422 | Up (0) | - |
|  |  |  | Down (3) | *rpsE; rplJ; rpmD* |
