# Supplementary material for: RNA-Seq-based transcriptome analysis of methicillin-resistant Staphylococcus aureus growth inhibition by propionate
Source: Front Microbiol. 2022 Dec 22;13:1063650. doi: 10.3389/fmicb.2022.1063650 (PMC9814166; doi:10.3389/fmicb.2022.1063650)
Supplement: Supplementary file 5 [file Table_5.DOCX]

**SUPPLEMENTARY TABLE 5 |** KEGG enrichment analysis of DEGs by NaP treatment.

| KEGG pathway | ID | P-value | No. of genes | Genes |
| --- | --- | --- | --- | --- |
| One carbon pool by folate | Saa00670 | 3.23×10^-2^ | 3 | *purH; purN; gcvT* |
| Ribosome | Saa03010 | 2.63×10^-2^ | 8 | *rpmB; rplY; rplR; rplF; rpmD; rpsE; rplJ; rplL* |
| Phosphotransferase system (PTS) | Saa02060 | 1.76×10^-2^ | 5 | *fruA; murP; ulaA; SAUSA300_RS01765; SAUSA300_RS01760* |
| Ascorbate & aldarate metabolism | Saa00053 | 1.12×10^-2^ | 3 | *ulaA; SAUSA300_RS01765; SAUSA300_RS01760* |
| Riboflavin metabolism | Saa00740 | 3.25×10^-3^ | 4 | *ribE; ribD; ribH; ribA* |
| Glycine, serine & threonine metabolism | Saa00260 | 9.17×10^-4^ | 8 | *gpmI; ilvA; thrB; gcvT; betB; hom; thrC; thrD* |
| Purine metabolism | Saa00230 | 8.44×10^-4^ | 10 | *arcC; purC; purS; purH; purN; purD; purL; purQ; purM; purF* |
| *Staphylococcus aureus* infection | Saa05150 | 2.33×10^-7^ | 15 | *aur; lukF-PV; lukS-PV; lukG; lukH; scn; hlgC; hlgB; sbi; hlgA; efb; scc; spa; chs; flr* |
