# Supplementary material for: RNA-Seq-based transcriptome analysis of methicillin-resistant Staphylococcus aureus growth inhibition by propionate
Source: Front Microbiol. 2022 Dec 22;13:1063650. doi: 10.3389/fmicb.2022.1063650 (PMC9814166; doi:10.3389/fmicb.2022.1063650)
Supplement: Supplementary file 6 [file Presentation_1.PPT]

## Slide 1
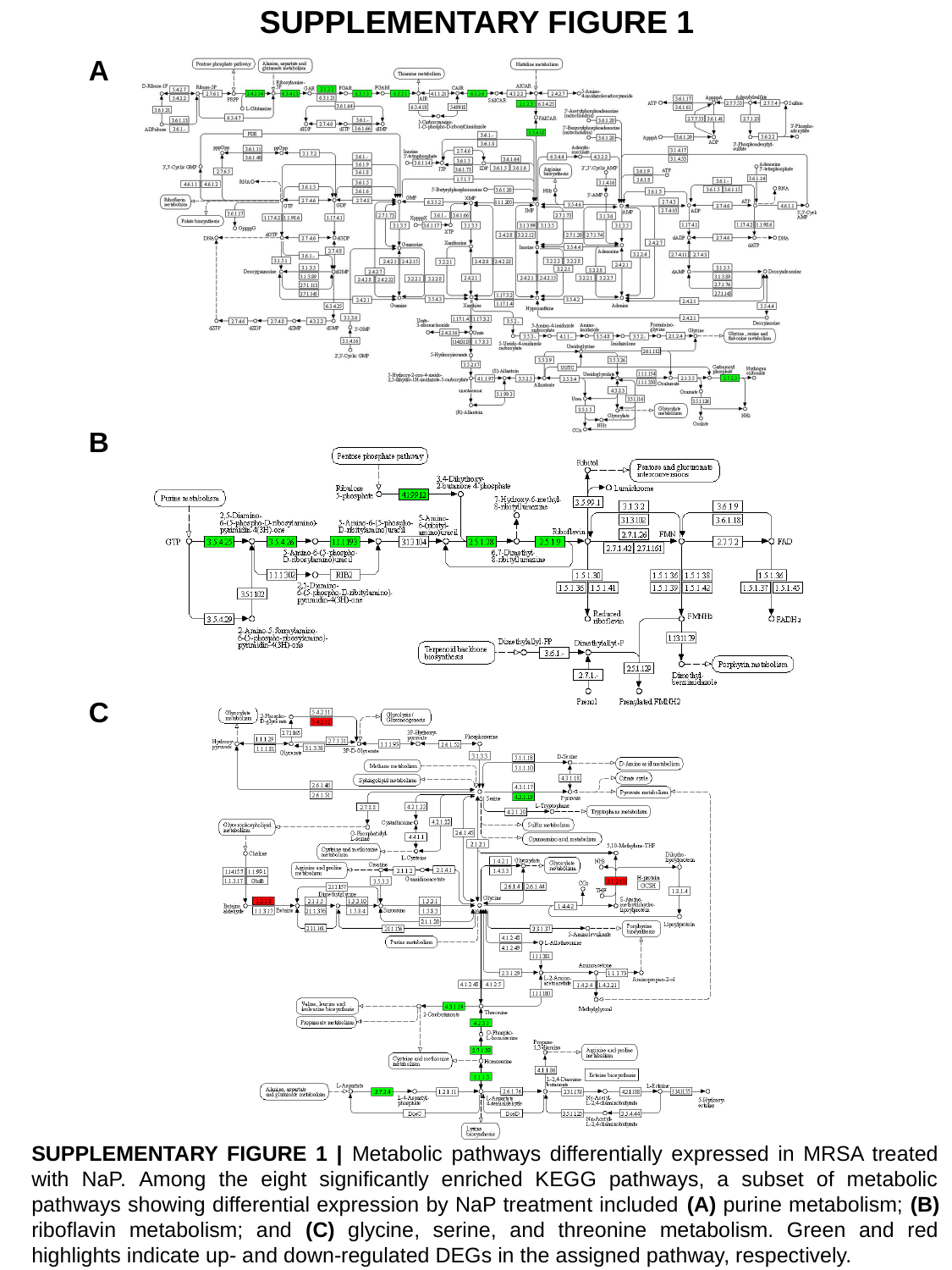

SUPPLEMENTARY FIGURE 1
A
B
C
SUPPLEMENTARY FIGURE 1 | Metabolic pathways differentially expressed in MRSA treated with NaP. Among the eight significantly enriched KEGG pathways, a subset of metabolic pathways showing differential expression by NaP treatment included (A) purine metabolism; (B) riboflavin metabolism; and (C) glycine, serine, and threonine metabolism. Green and red highlights indicate up- and down-regulated DEGs in the assigned pathway, respectively.
