# Supplementary material for: RNA-Seq-based transcriptome analysis of methicillin-resistant Staphylococcus aureus growth inhibition by propionate
Source: Front Microbiol. 2022 Dec 22;13:1063650. doi: 10.3389/fmicb.2022.1063650 (PMC9814166; doi:10.3389/fmicb.2022.1063650)
Supplement: Supplementary file 7 [file Presentation_2.PPT]

## Slide 1
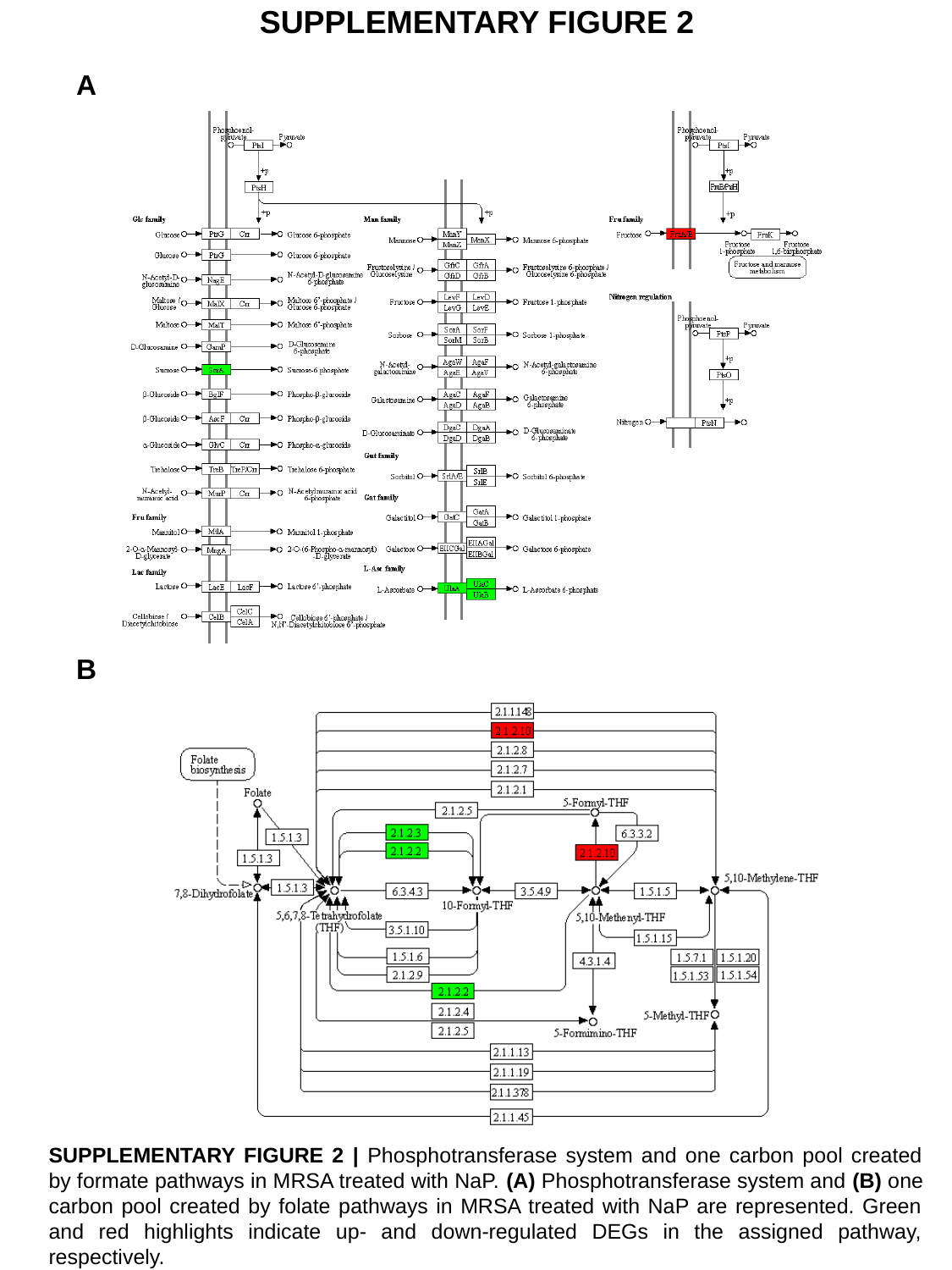

SUPPLEMENTARY FIGURE 2
A
B
SUPPLEMENTARY FIGURE 2 | Phosphotransferase system and one carbon pool created by formate pathways in MRSA treated with NaP. (A) Phosphotransferase system and (B) one carbon pool created by folate pathways in MRSA treated with NaP are represented. Green and red highlights indicate up- and down-regulated DEGs in the assigned pathway, respectively.
