# Supplementary material for: RNA-Seq-based transcriptome analysis of methicillin-resistant Staphylococcus aureus growth inhibition by propionate
Source: Front Microbiol. 2022 Dec 22;13:1063650. doi: 10.3389/fmicb.2022.1063650 (PMC9814166; doi:10.3389/fmicb.2022.1063650)
Supplement: Supplementary file 8 [file Presentation_3.PPT]

## Slide 1
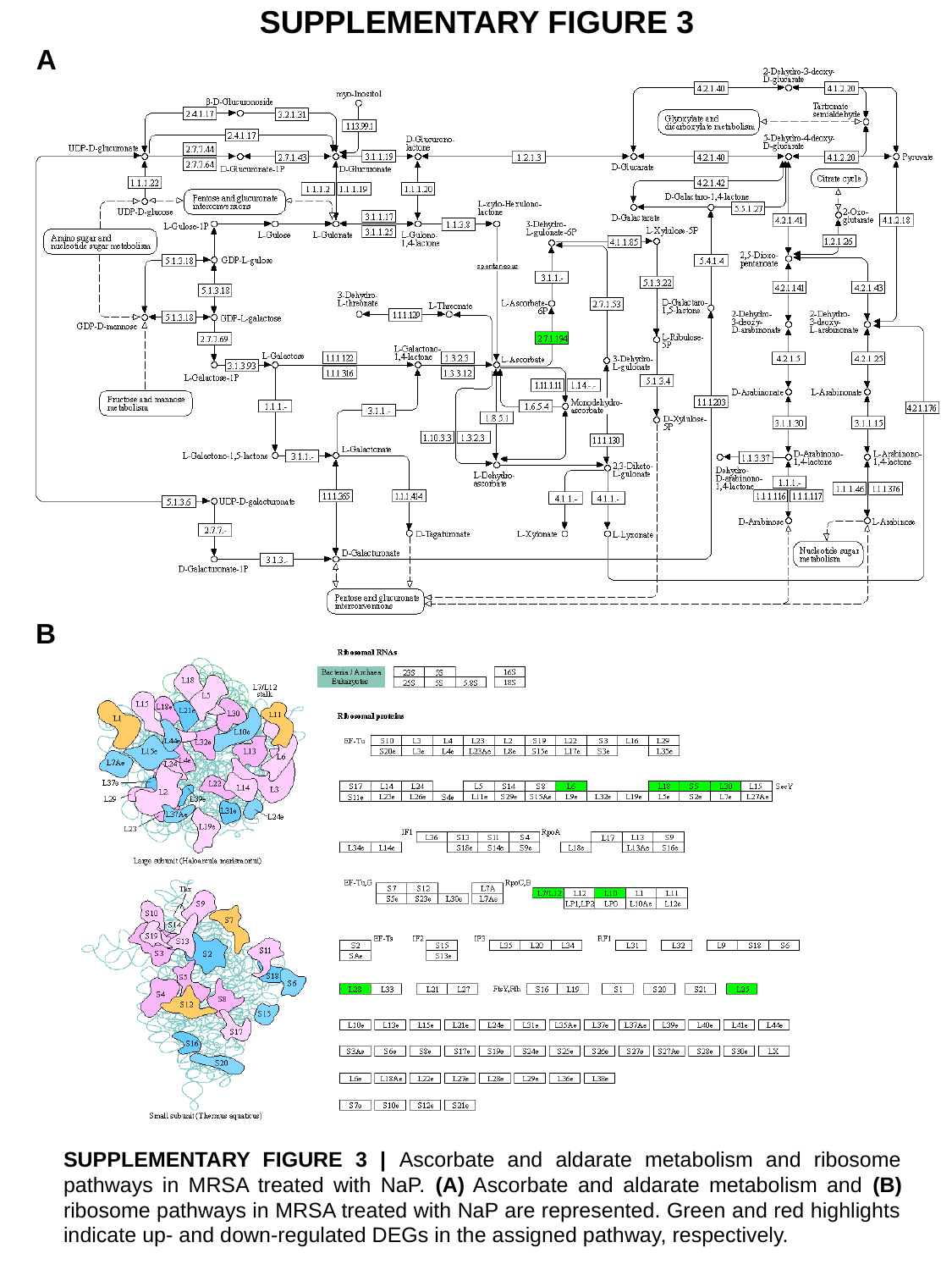

SUPPLEMENTARY FIGURE 3
A
B
Ribosome
SUPPLEMENTARY FIGURE 3 | Ascorbate and aldarate metabolism and ribosome pathways in MRSA treated with NaP. (A) Ascorbate and aldarate metabolism and (B) ribosome pathways in MRSA treated with NaP are represented. Green and red highlights indicate up- and down-regulated DEGs in the assigned pathway, respectively.
