# Supplementary material for: RNA-Seq-based transcriptome analysis of methicillin-resistant Staphylococcus aureus growth inhibition by propionate
Source: Front Microbiol. 2022 Dec 22;13:1063650. doi: 10.3389/fmicb.2022.1063650 (PMC9814166; doi:10.3389/fmicb.2022.1063650)
Supplement: Supplementary file 9 [file Presentation_4.PPT]

## Slide 1
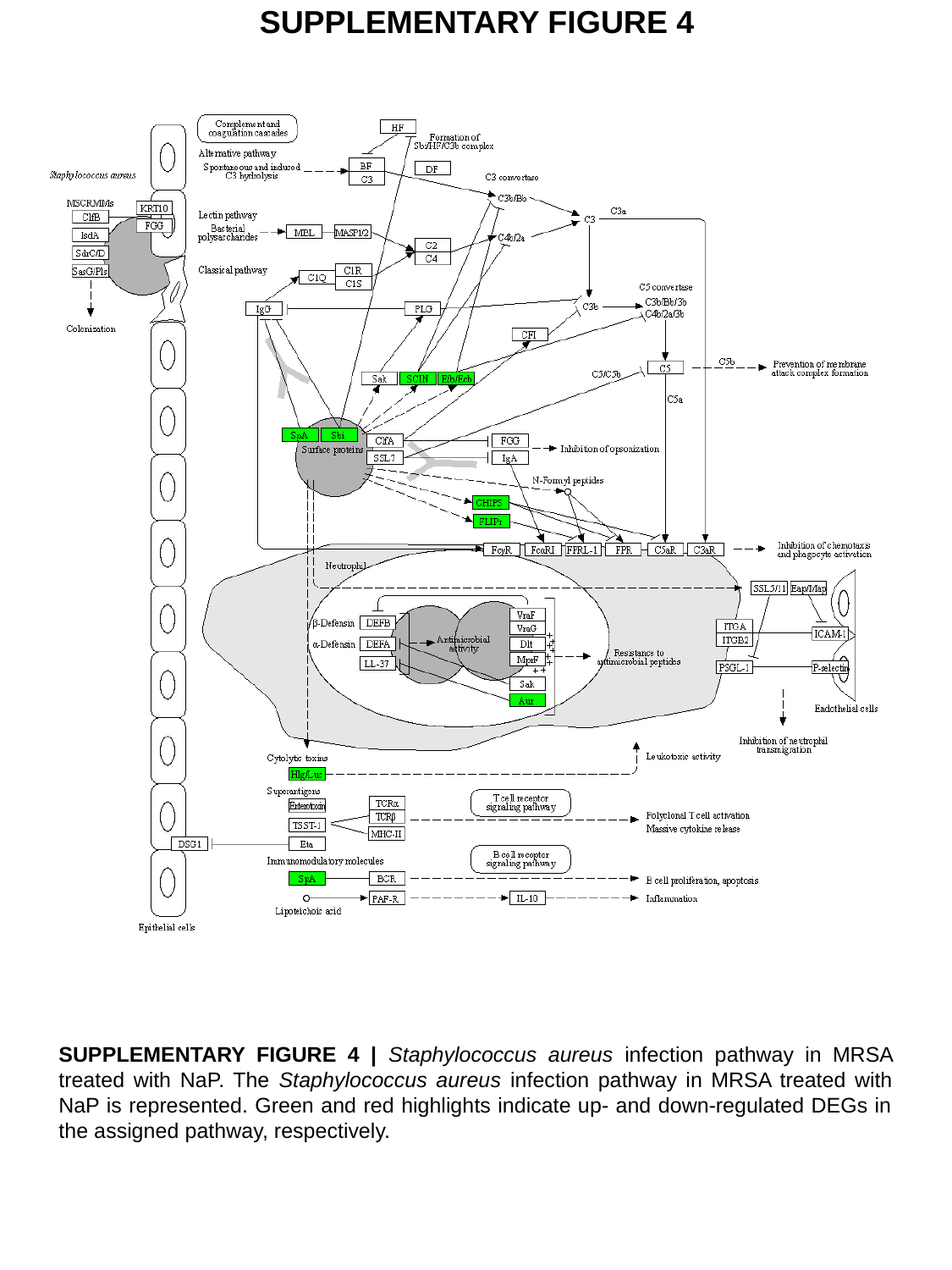

SUPPLEMENTARY FIGURE 4
SUPPLEMENTARY FIGURE 4 | Staphylococcus aureus infection pathway in MRSA treated with NaP. The Staphylococcus aureus infection pathway in MRSA treated with NaP is represented. Green and red highlights indicate up- and down-regulated DEGs in the assigned pathway, respectively.
